# Processes and therapeutic perspectives of acylation modifications of lysine and cysteine in tumors

**DOI:** 10.1186/s12964-026-02707-4

**Published:** 2026-02-02

**Authors:** Jialin Jiang, Jiabin Chen, Shuhang Huang, Yue Tian, Lanyu Liu, Jiahui Yao, Yuzhu Zhang, Can Jiang, Xingting Zhang, Na Han, Guang Shu, Gang Yin, Li Xian Yip, Kuoran Xing, David Tai Leong, Maonan Wang

**Affiliations:** 1 Department of Pathology, Xiangya Hospital, Xiangya School of Basic Medical Sciences, Central South University, Changsha, China; 2 National Clinical Research Center for Geriatric Disorders, Xiangya Hospital, Central South University, Changsha, China; 3 Department of Chemical and Biomolecular Engineering, College of Design and Engineering, National University of Singapore, Singapore, Singapore

**Keywords:** Lipidation, Acetylation, Succinylation, Myristoylation, Crotonylation, Malonylation, Glutarylation, Targeted nanomaterials

## Abstract

Acylation modification plays a crucial role in tumor progression through altering protein homeostasis and localization. However, a comprehensive summary of these processes is lacking. Protein acylation modifications are comprehensively summarized and categorized based on the amino acids that are modified. This review focuses on modifications of the lysine (acetylation, succinylation, crotonylation, malonylation, and glutarylation) and cysteine (myristoylation and palmitoylation) groups. The key enzymes involved in the occurrence and erasure of different modifications, as well as their effects on protein stability, cell localization, and tumor progression, are highlighted. The targeted delivery systems related to acylation modification are summarized, and currently available commercial inhibitors are also reviewed. Finally, acylation modifications with therapeutic potential in Phase I clinical trials are reviewed.

**Supplementary Information:**

The online version contains supplementary material available at 10.1186/s12964-026-02707-4.

## Background

Mechanistic studies of tumor progression are increasingly focusing on protein modification in which lipidation plays an important role. Protein lipidation involves covalent conjugation of lipid molecules with proteins, which alters the structural conformations and functional characteristics of proteins. The molecular architectures of various lipids, such as fatty acids, cholesterol, terpenes, and phospholipids, are fundamentally distinct, indicating differences in linkage modalities between lipids and proteins. Fatty acids predominantly associate with proteins via acyl group attachments. These bonds are relatively unstable, enabling protein S-acylation, a rare reversible lipid modification. The more common lipid acylation modifications are predominantly irreversible. The sole reactive group in cholesterol is 3β-hydroxyl, which typically forms ester bonds with terminal carboxyl groups of proteins [1]. Lipids can also conjugate with carbohydrates to generate glycolipid complexes, particularly glycosyl phosphatidy linositol anchors. Lipidation increases the lipophilicity of proteins as well as their global and regional hydrophobicities. Lipidation is a biochemical alteration that increases the affinity of modified proteins for the corresponding membrane structures or induces localized conformational changes, influencing their subcellular localization and functional activity.

Comprehensive reviews detailing acylation modification are lacking. Therefore, here, we review the protein lipidation literature to summarize its defining characteristics and classifications. We focus on modifications to lysine (acetylation, succinylation, crotonylation, malonylation, and glutarylation) and cysteine (myristoylation and palmitoylation) groups. Crotonylation can be mediated not only by specific enzymes but also occurs through non‑enzymatic reactions, thereby broadening the regulatory scope of protein modifications. This review emphasizes the elucidation of key enzymes involved in the establishment and removal of distinct modifications, along with their impacts on protein stability, cellular localization, and tumor progression. The targeted delivery systems related to acylation modification and currently available commercial inhibitors are summarized. Finally, the clinical evidence from Phase I trials investigating the application of these systems is summarized [2].

## Acylation modifications

Acylation involves the formation of amide bonds between fatty acids and either the terminal α-amino groups of polypeptide chains or the ε-amino groups of the side chains of lysine. Therefore, acylation occurs on lysine, which includes acetylation, succinylation, crotonylation, malonylation, and glutarylation or on cysteine, which includes myristoylation and palmitoylation [3].

Acylation modifications are classified into N-, O-, and S-acylation (Table 1). O-acylation involves the formation of ester bonds between fatty acids and hydroxyl groups of the side chains of serine. The sole form of O-acylation is O-palmitoylation, which rarely occurs. S-acylation is a rare reversible modification in which fatty acids conjugate with cysteine thiol groups via thioester linkages. However, these high-energy bonds are unstable under physiological conditions [4]. The predominant S-acyl group is palmitate (16:0), followed by stearate (18:0), oleate (18:1), arachidonate (20:4), and eicosapentaenoate (20:5). Palmitate accounts for 74% of S-acyl modifications, followed by stearate (22%) and oleate (4%). Some studies have inappropriately generalized all S-acylations as S-palmitoylations owing to the predominance of palmitate in S-acylation discovery [5]. Palmitoylations are classified into N-, S-, and O-palmitoylation, depending on the donor molecule specificity.

**Table 1:** Different types of acylation modification

Name	Bond	Example
N-acylation	Amide bond	N-acetylation
		Succinylation
		Myristoylation
		N-palmitoylation
		Crotonylation
		Malonylation
		Glutarylation
O-acylation	Ester bond	O-palmitoylation
S-acylation	Thioester bond	S-palmitoylation

S-palmitoylation is a post-translational modification (PTM) that occurs in the Golgi apparatus and involves catalysis by protein acyltransferases (PATs) containing the conserved distinctive Asp-His-His-Cys (DHHC) motif, termed DHHC-PATs. Catalysis is an acyl transfer reaction in which DHHC-PATs first bind palmitoyl-CoA to form an “enzyme-palmitoyl intermediate” and then transfer the palmitoyl group to the thiol group (-SH) of a specific cysteine on the target protein. This generates the modified protein and releases CoA–DHHC-PATs, regaining activity for repeated catalysis. The DHHC motif is a zinc finger domain, with the gene nomenclature *zinc finger DHHC* (*zDHHC*). Unlike S-acylation, N-acylation and O-acylations are irreversible modifications, whose effects on protein structure or location can directly contribute to pathological progression. The subsequent sections focus on acylation by building upon this mechanistic framework.

## Acylation modifications on lysine

The acylation modifications that occur on lysine include acetylation, succinylation, crotonylation, malonylation, and glutarylation.

### N-acetylation

Acetylation modifications of histones were discovered in 1963. The acetylation status of histones correlates with gene transcription activity. Hyperacetylation is generally associated with transcriptional activation, whereas hypoacetylation is linked to transcriptional silencing. However, the specific molecular mechanisms of these processes remained a black box for nearly three decades due to technological limitations. Allfrey et al. identified the lysine acetylation of histones in 1964 and suggested that this protein modification plays an important role in regulating transcription [6]. However, technological constraints have prevented lysine acetylation from being comprehensively characterized. Researchers have since been able to characterize lysine acetyltransferase/lysine deacetylase (KAT/KDAC) activities, expanding the scope of acetylation studies to include nonhistone substrates.

Acetylation is an enzymatic or nonenzymatic process during which acetyl groups are covalently conjugated to ε-amino groups of lysine residues or α-amino termini of proteins via transfer catalyzed by acetyltransferases [7]. Acetylation modifications are divided into N-terminal acetylation at the α-amino termini of proteins and lysine acetylation at ε-amino groups of lysine residues, depending on the location of the acetyl group transfer. Lysine acetylation is a reversible and dynamic PTM involved in regulating cancer processes via altering functional homeostasis and interfering with gene expression [8, 9]. The mechanisms underlying acetylation include (i) regulating disease-related genes through modifications of histone acetylation; (ii) acetylating the target gene that affects the disease; (iii) acetylating the upstream regulator of the target gene, directly affecting the disease; and (iv) affecting target gene in the disease by recruiting a gene to regulate the deacetylation of another gene.

**Fig. 1:**
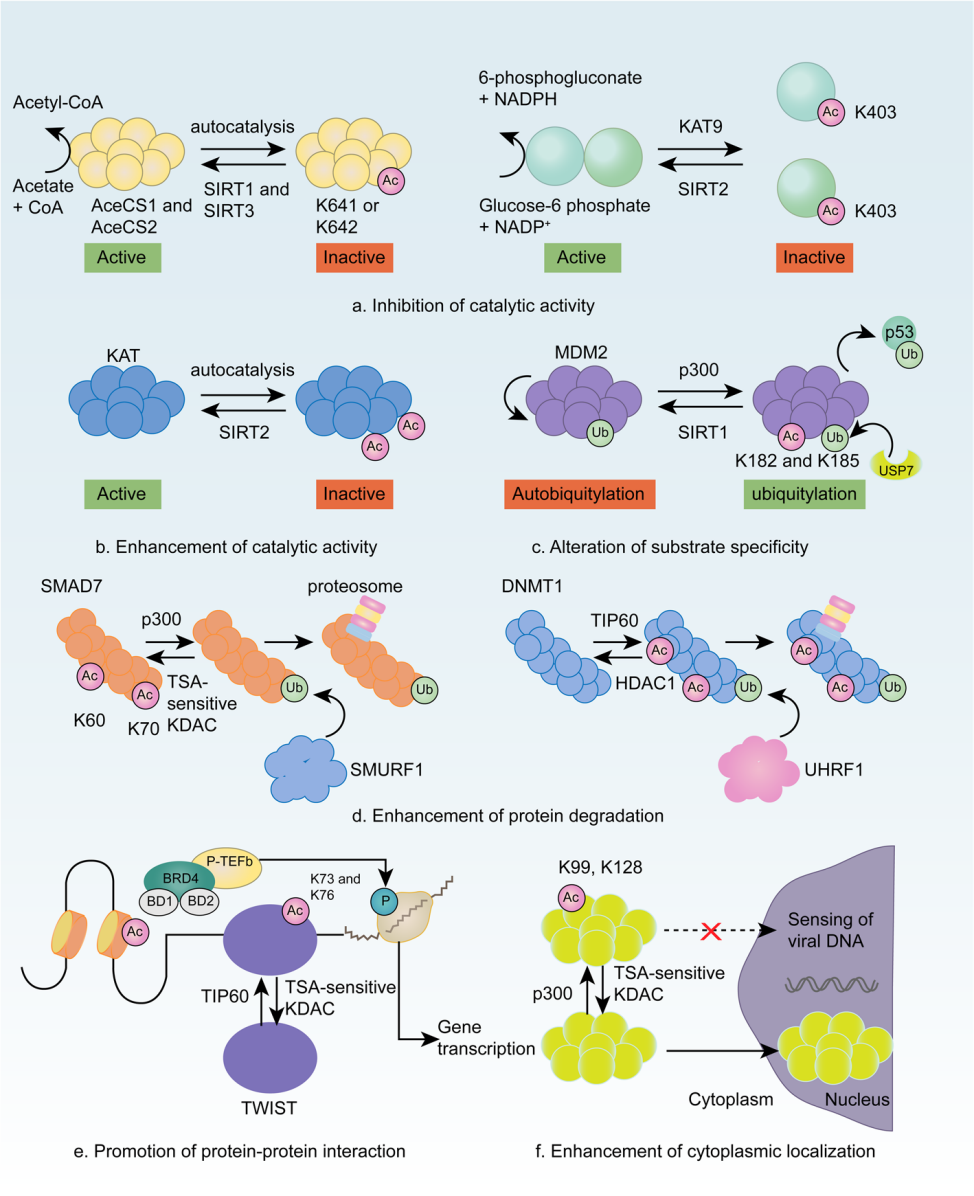
Pathways through which N-acetylation modifications occur. **a** Acetylation inhibits the catalytic activity of long-chain acyl-CoA dehydrogenase, which is restored via deacetylating sirtuin 3 (SIRT3). **b** Acetylation increases the phosphatase activity of mitogen-activated protein kinase (MAPK) phosphatase 1 (MKP1) and the interactions of MKP1 with MAPK p38, inhibiting MAPK signal transduction. **c** Acetylation changes the substrate specificity of E3 ubiquitin-protein ligase mouse double minute 2 homolog (MDM2) from self-ubiquitination to ubiquitination of the main substrate, p53. **d** Lysine acetylation regulates proteasome-dependent and -independent protein degradation. **e** Acetylating nonhistone proteins promotes or inhibits protein–protein interactions. **f** Viral infection triggers P300-dependent acetylation of the nuclear localization signal of the viral DNA sensor interferon-gamma-induced protein 16 (IFI16), promoting the cytoplasmic localization of IFI16. KDAC, lysine deacetylase; TSA, trichostatin A [11]

N-acetylation is one of the most common protein modifications in eukaryotes [10]. N-terminal acetylation involves the transfer of an acetyl group to the α-amino group of the first amino acid residue at the N-terminus of the protein. N-terminal acetylation is catalyzed by N-terminal acetyltransferases (NATs), with acetyl-CoA serving as the obligatory acyl donor. Protein N-acetylation is linked to protein stability and membrane binding. The cellular signaling pathways associated with N-protein acetylation, as well as the molecular mechanisms underlying its regulation, are examined in this review. The six main functions of protein acetylation modifications include inhibiting enzyme activity, increasing enzyme activity, altering enzyme–substrate specificity, regulating protein degradation, promoting protein–protein interactions, and regulating the subcellular protein localization (Fig. 1).

Acetylation studies generally involve (1) determining whether the target protein is acetylated, (2) screening for acetylases/deacetylases, (3) identifying acetylation sites, (4) determining the effect of acetylation on the target protein, and (5) exploring the biological functions of the acetylation of the target protein. Proteomic analyses suggest that nonhistone proteins frequently undergo acetylation and that these proteins comprise the main portion of acetylated proteins within mammalian cells. The acetylation of nonhistone proteins is involved in vital physiological and illness-associated cellular processes, such as gene transcription, DNA damage restoration, cell division, signal transmission, protein conformation, autophagy, and metabolic activities.

KATs and KDACs are crucial in conventional protein acetylation. KDACs are further categorized into zinc ion-dependent HDACs and NAD^+^-dependent sirtuin deacetylases [11]. However, no NAD + -dependent deacetylases have been identified that act on the N-terminus. Thus, N-acetylation is often considered irreversible (Table 2). N-acetylation alters the chemistry of the N-terminus of proteins by neutralizing the electric charge, generating new hydrogen bond receptors, modifying the nucleophilic and basic properties of the α-amino nitrogen, and increasing protein hydrophobicity and size [10]. These changes in chemical properties exert a range of biological effects on protein functionality, such as protein stability, degradation rate, folding conformation, complex assembly, and subcellular localization. Knockouts of individual eukaryotic NATs exhibit distinct phenotypes, likely reflecting dysregulation of their client proteins [40].

**Table 2:** Summary of enzymatic and cellular features of mammalian sirtuins

Sirtuin	Enzymatic Function	Localization	Targets	Experimental Types	Cellular Functions	Reference
SIRT1	Deacetylase	Nucleus, cytoplasm	H3K9, H3K14, H4K16,	Nucleosome assay	Energy homeostasis, differentiation, neuroprotection, DNA repair	[12] [13] [14] [15] [16]
			P53, FOXO3a, NBS1, BCRA1, SUV39H1, PGC-1α, PCAF	Synthetic peptide		[17] [18], [19] [20] [21] [21] [22]
SIRT2	Deacetylase	Nucleus, cytoplasm	H4K16	Nucleosome assay	Cell cycle regulation, tubulin deacetylation	[23]
			α-Tubulin, FOXO	Synthetic peptide		[24] [25]
SIRT3	Deacetylase	Mitochondria	AceCS2, JNK2, MnSOD	Synthetic peptide	ATP production, mitochondrial homeostasis regulation, fatty acid oxidation	[26], [27], [28]
SIRT4	ADP-ribosyl transferase, lipoamidase	Mitochondria	GDH, ANT	Synthetic peptide	Insulin secretion	[29], [30]
SIRT5	Deacetylase, desuccinylase, demalonylase	Mitochondria	CPSI,	Synthetic peptide	Urea cycle	[31]
SIRT6	Deacetylase, ADP-ribosyl transferase, deacylase	Nucleus	H2BK12, H3K27, H3K9, H3K56, H3K18, KAP1, PGC-1α, GCN5	Nucleosome assay Synthetic peptide	Glucose homeostasis, DNA repair, telomeric function, cellular differentiation, mitosis and meiosis, cancer	[32] [33] [12] [12] [34] [35] [36] [37]
SIRT7	Deacetylase	Nucleus (nucleolus)	H3K18	Nucleosome assay	RNA Pol-I-dependent transcription, DNA repair	[34]
			EIA, Smad6	Synthetic peptide		[38] [39]

#### HDAC classification and characteristics

##### Class I HDACs (HDAC1/2/3/8)

Class I HDACs localize in the nucleus, where these HDACs typically function as core components of multiprotein complexes (e.g., Sin3, NuRD, and CoREST) and primarily target histones (e.g., H3 and H4) for deacetylation. Class I HDACs thus regulate transcriptional silencing and cell cycle progression. For example, HDAC3 interacts with nuclear receptor corepressors (NCOR and SMRT) to modulate inflammation and metabolic regulation.

##### Class II HDACs (IIa: HDAC4/5/7/9; IIb: HDAC6/10)

Class IIa HDACs shuttle between the nucleus and cytoplasm in response to calcium signaling (e.g., via CaMK activation). Class IIa HDACs regulate processes such as muscle differentiation. For example, HDAC4 suppresses MEF2. Class IIb members such as HDAC6 are mainly cytoplasmic and possess unique tubulin deacetylation functions, participating in forming stress granules and clearing protein aggregates.

##### Class III HDAC (Sirtuins)

Sirtuins are NAD-dependent Class III HDACs with many enzymatic activities, such as deacetylation and ADP-ribosylation. Sirtuins, including SIRT1–7, are closely associated with metabolism, aging, and cancer. Critically, their enzymatic activity is directly governed by intracellular NAD⁺ concentrations, which in turn determine their capacity to orchestrate signaling pathways involved in these processes.

SIRT1 deacetylates nuclear histones (H3K9 and H4K16) and nonhistone proteins (e.g., p53 and FOXO3a) to regulate stress responses and lifespan. SIRT2 is predominantly cytoplasmic and targets α-tubulin and CDK9, playing roles in cell cycle checkpoints and mitosis. SIRT3–5 localize in the mitochondria. SIRT3 deacetylates metabolic enzymes, such as isocitrate dehydrogenase 2 and superoxide dismutase 2, and participates in modulating oxidative stress. SIRT4 exhibits ADP-ribosyltransferase activity, inhibiting glutamine metabolism. SIRT5 catalyzes desuccinylation/demalonylation, affecting the urea cycle. SIRT6 localizes in the nucleus and deacetylates H3K9/H3K56 to regulate DNA repair and glucose metabolism, suppressing the Warburg effect. SIRT6 plays two opposite roles in cancer, suppressing liver cancer tumors but exhibiting oncogenic activity in prostate cancer. SIRT7 also localizes in the nucleus and targets RNA polymerase I and histone H3K18, promoting ribosome biogenesis and cancer cell proliferation.

The traditional free peptide substrates in HDAC/sirtuin activity assays may not accurately reflect the true activity of the enzyme in the chromatin environment. Nucleosomes or nucleosome arrays can be used as substrates to more realistically simulate physiological conditions [41]. The substrate environment strongly affects the results when screening for inhibitors or activators. Owing to the lack of a higher-order chromatin structure, free peptides fail to reflect enzymatic kinetic characteristics accurately, whereas the catalytic efficiency of HDAC/sirtuin is more accurately assessed when using nucleosome substrates [42]. The activity of certain HDACs and sirtuins, such as HDAC3 and SIRT6, respectively, on nucleosomes widely differs from that in free peptide systems, suggesting that substrate models that are more physiologically relevant should be prioritized when developing drugs [43]. These studies support shifting to nucleosome-based detection systems for epigenetic drug screening to improve both the biological relevance and translational potential of the data.

##### Class IV HDAC (HDAC11)

HDAC11 is structurally similar to other HDACs but is functionally distinct HDAC11 mainly functions to regulate fatty acid metabolism and immune tolerance, for example, the expression of interleukin-10. Notably, in vitro assays employing synthetic peptides may not fully recapitulate the physiological activity of enzymes on nucleosomal substrates within a chromatin context [41].

Histone acetylation alters the DNA charge, opening euchromatin. Therefore, histone acetylation leads to more active gene transcription, which may play a role in cancer development [44]. KAT6A modifies SMAD3 via acetylation at K20 and K117, which disrupts the interaction between SMAD3 and tumor suppressors and increases the oncogenic activity of SMAD3. Increased SMAD3 activity increases breast cancer stem-like cell stemness, leads to recruitment of myeloid-derived suppressor cells, and results in metastasis in triple-negative breast cancer [45]. Histone acetylation can also impede tumor progression. For example, histone acetylation facilitates the expression of p300, which increases the expression level of the catechol-O-methyltransferase (*COMT*) gene as well as the metabolism of COMT to estrogen and hinders the progression of breast cancer [44].

p300 expression is upregulated by histone deacetylation, and HDAC inhibitors are powerful tools for treating cancer. This class of inhibitors acts on the histones that wrap DNA and functions by controlling how tightly or loosely DNA is wrapped around histones. HDACs cause DNA to wrap more tightly around histones via histone deacetylation, hindering the access of gene transcription factors to DNA. This suppresses the expression of proteins related to cell differentiation and leads to cell cycle arrest, tumor immunity, and apoptosis in damaged cells. All of these processes are closely associated with tumor development.HDAC inhibitors inhibits tumor development through various pathways, such as hyperacetylating histones, and activates a variety of transcription factors.HDAC inhibitors can thus inhibit cancer via altering target gene expression, blocking the cell cycle, and promoting tumor cell senescence [46].

In tumor immunity, HDAC inhibitors strengthens the immunogenicity and antigen-presenting capacity of tumor cells by stimulating the natural killer (NK) group 2 member D (NKG2D) signaling pathway.HDAC inhibitors thus promotes the expression of tumor antigens, upregulating MHC class I and II molecules, along with their associated antigen-processing mechanisms, costimulatory molecules, and NK-cell-activating ligands.HDAC inhibitors also reduces NK cell activation and damage to tumor cells. Practical HDAC inhibitors treatments include vorinostat, which is used to treat cutaneous T-cell tumors; belinostat, which is used to treat peripheral T-cell tumors; and chidamide and romidepsin, which are effective at treating cutaneous and peripheral T-cell tumors. Additionally, histone acetyltransferase (HAT) inhibitors, HAT activators, and HDAC activators are medications used in breast cancer therapy [44]. The deacetylase inhibitors considered in clinical trials are summarized in Table S1.

### Succinylation

Succinylation involves active and negatively charged PTM of proteins via covalent binding of the succinyl groups of succinyl donors to lysine residues. The positive charge of residues is neutralized, creating a net negative charge at physiological pH and thus strongly influencing the structure and function of proteins [47]. Succinylation adds more mass to lysine residues than do most other PTMs. Therefore, the changes in charge and mass caused by succinylation may markedly affect protein function.

**Fig. 2:**
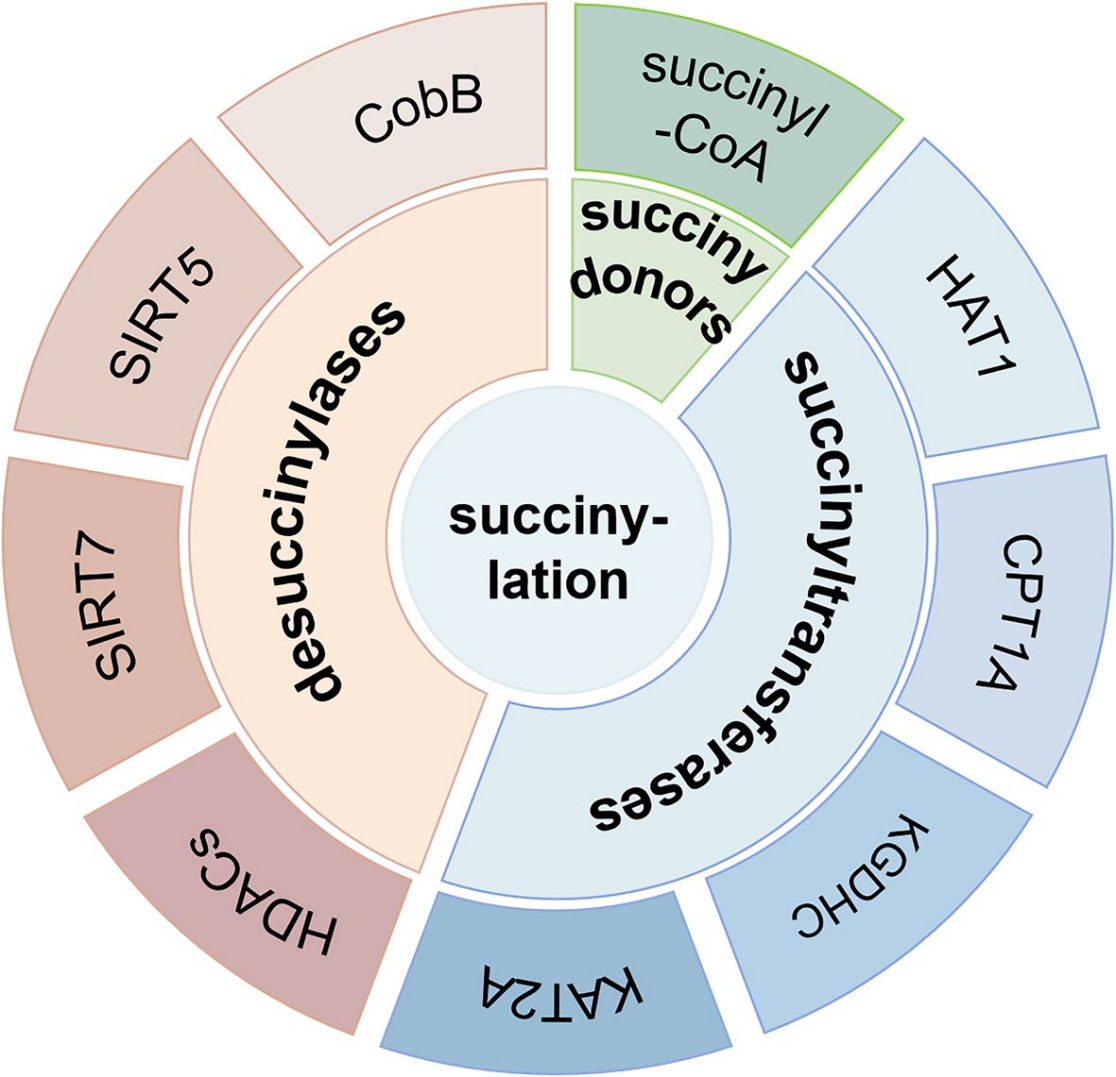
Succinyltransferases, desuccinylases, and succinyl donors regulate succinylation levels. Succinyltransferases include HAT1, CPT1A, KGDHC, and KAT2A. Desuccinylases include HDACs, SIRT7, SIRT5, and CobB. Succinyl-CoA is the main succinyl donor [68]

Succinylation occurs in many organisms, such as *Escherichia coli* and *Saccharomyces cerevisiae*, human cells, and mouse liver tissue [47, 48]. The succinylation profile is markedly heterogeneous across different subcellular locales. Succinylation in the cytoplasm mainly occurs in the mitochondria [49, 50]. More than one-third of the nucleosomes in the nucleus contain lysine succinylation markers. Succinylation sites mainly concentrate in the promoter region [49], suggesting that succinylation participates in regulating gene transcription. The formation of most tumors is related to succinate dehydrogenase (SDH) mutations. SDH is a respiratory enzyme involved in the tricarboxylic acid cycle (TCA) and electron transport chain. Succinyl coenzyme A is the main donor for succinylation and an intermediate TCA metabolite [51], suggesting that succinylation is closely related to energy metabolism. Succinylation is involved in many biological processes through regulating protease activity and gene expression. Succinylation regulation is determined by “writers”, “erasers”, and “readers” (Table 3). Succinylation levels are mainly regulated by succinyl donors, succinyltransferases, and desuccinylases (Fig. 2), which affect tumor development by regulating different mechanisms. The following section discusses the research progress on succinylation regulatory enzymes and succinylation modifications in tumors.

**Table 3:** Succinylation writers, erasers, and readers

Enzyme function	Enzyme	Substrate	Experimental Types	Ksucc site	Reference
Writer	KGDHC/ KAT 2A	Histone 3	Nucleosome assay	K79	[52, 53]
	CPT 1A	LDHA S100A10	Synthetic peptide	K222 K47	[54, 55] [56, 57]
	HAT 1	Histone 3	Nucleosome assay	K122	[52, 58]
Eraser	SIRT 5	GLS	Synthetic peptide	K164 K158	[31, 59, 60]
Reader	SIRT 7 HDAC1–3 GAS 41	SHMT 2 Histone 3 PRMT 5 Histone 3 Histone 3	Nucleosome assay	K280 K122 K387 K14 K23 K122	[61, 62] [52, 63] [64, 65] [52, 66] [52, 67]

#### Succinylation regulatory enzymes

##### Succinyl donors

The main succinyl donor is succinyl coenzyme A, the level of which regulates lysine succinylation [47]. The main sources of succinyl coenzyme A are the TCA cycle, amino acid metabolism, and fatty acid oxidation [69]. Succinyl coenzyme A is an important intermediate in the TCA cycle [70], where a change in its concentration affects the succinylation level and tumor progression. An imbalance in related enzymes in the TCA cycle may lead to cancer [71].

##### Succinyltransferases

Succinyltransferases are mostly involved in energy metabolism in cells. The α-ketoglutarate dehydrogenase complex (KGDHC) functions as a transferase in α-ketoglutarate-dependently succinylating mitochondrial proteins and mediating protein succinylation [72]. Purified KGDHC succinylates a variety of proteins and affects related enzymes in the TCA, playing an important role in regulating gene expression levels [72]. KGDHC catalyzes the conversion of α-ketoglutaric acid to succinyl coenzyme A as a donor for succinylation [73]. Lysine acetyl-coenzyme A transferase 2 A (KAT2A, also known as GCN5) is the first HAT identified as being related to transcription. KAT2A can be used as a histone succinyltransferase to transfer succinyl groups to histone H3 lysine 79 (H3K79), which is key for regulating cellular gene expression [74]. Additionally, KGDHC couples with KAT2A in the promoter region of the nuclear gene to obtain local succinyl coenzyme A, allowing H3K79 succinylation with less succinyl coenzyme A than when the KGDHC-KAT2A collaborative system is not formed [75]. KAT2A and the α-ketoglutarate (α-KG)DH complex are closely related to tumor proliferation and growth [75].

Carnitine palmitoyltransferase (CPT1A) exhibits the same lysine succinyltransferase activity in vivo and in vitro [76]. CPT1A mediates the succinylation of the lactate dehydrogenase (LDHA) K222 site [77]. CPT1A independently functions as a CPT1A and succinyltransferase, regulating enzyme and substrate metabolism. Additionally, CPT1A acts as a transferase during S100A10 succinylation at lysine residue 47 (K47), which is related to cancer cell invasion and metastasis [78]. HAT1 regulates protein acetylation and is a succinyltransferase that mediates histone and nonhistone succinylation. HAT1 likely regulates the succinylation of histone 3 lysine 122 (H3K122) and nonhistone phosphoglycerate mutase 1 (PGAM1) K99. H3K122 succinylation contributes to epigenetic and gene expression regulation in cancer cells. An increase in PGAM1 activity stimulates glycolytic flux in cancer cells and plays a key role in promoting tumor progression [79].

##### Desuccinylases

Most desuccinylases are based on deacetylases, such as CobB, SIRT5, SIRT7, and class I HDACs (HDAC1–3). CobB is a bifunctional enzyme with lysine desuccinylation and deacetylation activities and was the first identified prokaryotic desuccinase. SIRT5 desuccinylates Cu/Zn SOD1 at K123 [80]. Glutaminase (GLS) affects cancer cell proliferation and promotes tumorigenesis through desuccinylation of lysine 164 (K164) and lysine 158 (K158) [81, 82]. SIRT5 regulates the desuccinylation of serine hydroxymethyltransferase 2 (SHMT2) K280, activates SHMT2, and subsequently affects the growth of tumor cells [83]. Mutations in SDH complex subunit A (SDHA) lead to the loss of cellular SDH activity, resulting in the accumulation of succinate. SIRT5 mediates SDHA K547 desuccinylation and affects tumor development [84].

Pyruvate kinase M2 (PKM2) is an important metabolic kinase of the Warburg effect, and PKM2 overexpression promotes tumor progression. SIRT5 mainly mediates PKM2 desuccinylation at lysine 498 (K498) and promotes lung cancer [85]. SIRT5 succinylates citrate synthase at K393 and K395, influencing cancer progression [86]. SIRT7 also exerts a desuccinylation effect, mediates H3K122 desuccinylation, and plays an important role in repairing DNA damage [87]. Lysine residue 387 (K387) of protein arginine methyltransferase 5 (PRMT5) is another succinylation site. SIRT7 catalyzes the desuccinylation of this site, facilitating reprogramming of lipid metabolism as well as affecting cancer cell proliferation and metastasis [88]. Histone desuccinylation, particularly promoter desuccinylation, is mainly catalyzed by HDAC1–3 rather than SIRT family proteins, suggesting that HDAC activity strongly correlates with gene transcription activity. The main desuccinylation sites are probably H3K14 and H3K23 [89]. Notably, in vitro assays employing synthetic peptides may not fully recapitulate the physiological activity of enzymes on nucleosomal substrates within a chromatin context [41].

#### Succinylation and tumor metabolic reprogramming

Succinylation plays a crucial role in cancer occurrence and development. The most important role of succinylation is influencing the metabolic reprogramming of tumors, which affects protein structure as well as function and thus glucose, lipid, and amino acid metabolism [90, 91].

##### Succinylation and glucose metabolism reprogramming

Cellular metabolism is the main difference between tumors and normal tissues in tumorigenesis. The effects of lysine succinylation on glucose metabolism in tumor cells are mainly observed in glycolysis, the TCA cycle, and the pentose phosphate pathway [92]. The metabolic changes that occur in cancer cells are known as the Warburg effect, which causes cancer cells to use glycolysis-generated energy, even under sufficient oxygen conditions, allowing cancer cells to rapidly proliferate with limited nutrition. The Warburg effect is regulated by protein succinylation [93, 94]. Specifically, phosphoglycerate kinase 1 (PGK1) and PKM2 are key enzymes involved in glycolysis, and their overexpression in tumor cells promotes tumorigenesis. PGK1 succinylation and desuccinylation affect PGK1 expression and activity in tumor cells. PGK1 is an ATP-producing enzyme involved in glycolysis, the overexpression of which affects the metabolic reprogramming induced by MYC and promotes tumor growth via enhancing glycolysis and the Warburg effect [95]. PGK1 overexpression thus shortens the survival of patients with tumors, suggesting that succinylation affects the PGK1 expression level and tumor progression [96, 97].

Glucose is first metabolized to glucose-6-phosphate (G6P), which undergoes glycolysis to produce pyruvate, under normal physiological conditions. However, cancer cells preferentially express the pyruvate kinase M2 subtype (PKM2), which exists as a low-activity dimer. The inactivated dimer PKM2 causes the accumulation of glycolysis intermediates and the transfer of G-6-P molecules to the pentose phosphate pathway, which helps tumor cells mitigate damage due to oxidative stress [97]. Decreased PKM2 activity leads to the transfer of more glucose to the pentose phosphate pathway and enhances the ability of tumor cells to resist oxidative stress. PKM2 succinylation at K498 enhances PKM2 activity and inhibits cell proliferation, whereas SIRT5 promotes tumor growth by preventing succinylation inhibitory activity at this site [85]. PKM2 succinylation at the K433 site promotes PKM2’s mitochondrial translocation, and PKM2 inhibits ubiquitination-dependent degradation binding that would affect voltage-dependent anion channel protein 3 (VDAC3) and stabilizes the outer mitochondrial membrane VDAC3. These processes increase the survival rate of tumor cells [98].

Succinyl coenzyme A is an intermediate in the TCA cycle, serving as a reactant and product in different steps of the cycle. Two of the ten steps in the TCA cycle are directly related to succinyl coenzyme A. The α-ketoglutarate dehydrogenase complex catalyzes the conversion of α-ketoglutarate, CoASH, and NADH to succinyl coenzyme A, NADH, and CO_2_, respectively. This step is irreversible, and α-ketoglutaric acid levels may be depleted because of lipid synthesis, affecting the production of succinyl coenzyme A [53]. The succinyl coenzyme A produced in this step continues to react with succinic acid and coenzyme A under the catalysis of succinyl coenzyme A synthase. Succinyl coenzyme A and succinic acid production and metabolism must therefore be balanced in the TCA cycle to maintain the cycle's metabolic homeostasis.

Succinyl coenzyme A synthetase ADP-forming subunit β (SUCLA2) participates in succinyl coenzyme A metabolism and regulates GLS K311 succinylation levels [99], which affects GLS activity and conformation. These increase tumor cell survival and proliferation. Therefore, changes in succinyl coenzyme A concentration affect tumor development by regulating tumor metabolism or succinyl modification levels. Furthermore, the balance between the production and metabolism of succinyl coenzyme A in the TCA cycle contributes to regulating succinylation in the glucose metabolism activated by tumors and thus the development of tumors [100]. Additionally, increased succinylation of the K48 and K140 sites of fructose diphosphate aldolase B and of the K6 site of transketolase in the N2 stage of the pentose phosphate pathway promotes cancer cell metastasis to lymph nodes [56].

##### Succinylation and reprogramming of amino acid metabolism

Tumor metabolic reprogramming involves reprogramming amino acid metabolism, such as glutamate and serine metabolism, in addition to glucose metabolism. Glutamine, the most abundant amino acid in the body, is an important component of tumor metabolic reprogramming as it participates in metabolizing nonessential amino acids, thereby providing energy for tumor development [101]. Glutamine supports anabolic processes in cancer cells and promotes cancer cell proliferation [58, 102]. GLS metabolizes glutamine into glutamic acid, which participates in synthesizing glutathione and enters the TCA cycle. The overactivated carcinogenic signal transduction in tumor cells upregulates GLS and SIRT5 [102]. SIRT5 mediates the desuccinylation of the K158 and K164 sites. SIRT5 knockdown lowers GLS levels. SUGLA2 interacts with GLS at the K311 site to regulate K311 succinylation. p38 mediates SUCLA2 phosphorylation under oxidative stress, which dissociates SUCLA2 from GLS and enhances K311 succinylation. GLS succinylation at K311 enhances K311 activity and provides energy for tumorigenesis.

Serine, the main source of one-carbon units in the body, links amino acid metabolism to the biosynthesis of nucleic acids and other substances, playing a role in tumor development. Serine is metabolized to glycine by SHMT2 [59]. SHMT2 succinylation at the K280 site reduces SHMT2 activity, and excessive succinylation at K280 inhibits cell proliferation and tumor growth. SIRT5 desuccinates SHMT2 at the K280 site, and this increases the catalytic activity of serine, leading to tumor cell proliferation and tumor progression.

##### Succinylation and lipid metabolism reprogramming

Cancer cells use lipid metabolism to support their rapid proliferation, survival, migration, invasion, and metastasis [60, 103]. Sterol regulatory element-binding protein-1 (SREBP-1) plays a central role in lipid metabolism. SREBP imbalance can drive malignant tumor growth [62, 104]. PRMT5-induced methylation prevents phosphorylation of SREBP1a, which leads to the dissociation of SREBP1a with Fbw7 (FBXW7). Thus, SREBP1a evades degradation via the ubiquitin–proteasome pathway. Stably methylated SREBP1a promotes lipid synthesis and accelerates cancer cell growth [105]. However, PRMT5 can be succinylated at K387, which affects the activity of PRMT5 as a methyltransferase. SIRT7 acts as a desuccinylase to desuccinylate PRMT5 at K387, degrading SREBP1a and strongly promoting lipid metabolism reprogramming, tumor growth, and metastasis in cancer cells. Table 4 summarizes the types of cancers involving succinylation and the sites at which succinylation occurs

**Table 4:** Tumor-related proteins that regulate succinylation modifications

Tumor	Proteins	Factors regulating succinylation	Sites	Mechanism	References
Breast cancer	GLS IDH 2	SIRT5	K169	Inhibits GLS degradation	[31, 63, 81] [106, 107]
Gastric cancer	S100A10	CAPT 1 A, SIRT5	K47	Inhibits protease degradation	[78]
	LDHA	CAPT1A	K222	Promotes cell invasion	[77, 108]
Hepatocellular carcinoma	ACOX1	SIRT5	——	Decreases enzyme activity and affects reactive oxygen species	[66]
	PGAM 1	HAT1	K99	Promotes tumor progression	[65]
Renal cell carcinoma	SDHA	SIRT5	K547	Affects SDHA activity	[84]
	PDHA 1	SIRT 5	K351	Regulatory metabolic path	[94, 109]
	HIF1, HIF2	CAP1A	——	Affects lipid droplet formation	[94, 110] [111]
Pancreatic ductal adenocarcinoma	14–3-3 β	KAT2A	K79	Promotes glycolysis	[112, 113]
	GLS	SIRT5	K311	Regulates succinyl-CoA concentration	[99]
Colorectal cancer	SHMT 2	SIRT5	K280	Promotes cell invasion	[83]
	PKM 2	SIRT5	K311	Affects macrophages	[114, 115]
	GLUD 1	SIRT5	——	Involved in glutamine metabolic recombination	[116]
	Citrate synthase	SIRT5	K393/K395	Promotes cell proliferation	[86]
Lung Cancer	SOD1	SIRT5	K123	Affects reactive oxygen species and stimulates cell growth	[80]

#### Crosstalk of succinylation with other PTMs

Most succinylation sites also act as acetylation sites, and lysine sites can be modified by several different acylations [48]. Lysine acetylation and succinylation exhibit similarities and differences. Compared with succinylation, acetylation weakly affects protein structure and charge. Lysine acetylation and succinylation play synergistic roles in regulating many cancers, such as breast cancer [117]. Malonylation and succinylation modify acid lysine and are regulated by SIRT5 [100]. However, malonylation and succinylation regulate different factors and proteins in different pathways [118].

##### Crosstalk with other nonacylated PTMs

Succinylated lysine possesses two negative charges that are larger than those of methylated lysine. Protein methylation has been extensively studied. Methylation is mainly regulated by methyltransferases and demethylases and participates in gene expression and cancer-related pathways [119]. Methylation and succinylation together participate in tumor occurrence and development [88]. Ubiquitination is a common PTM that maintains cell homeostasis. An imbalance between ubiquitination and deubiquitination leads to tumorigenesis [120]. Succinylation and ubiquitination are involved in regulating innate immunity [121]. The ubiquitination and deubiquitination of proteins related to tumor metabolism regulate the signaling pathways of tumor metabolism, suggesting the production of synergistic or antagonistic effects with succinylation [122].

### Crotonylation

Crotonylation is a PTM that occurs on the lysine residues of proteins, formed via the covalent linkage of a crotonyl group to an ε-amino group of lysine [123]. This modification was identified in histones in 2011 and complements other epigenetic mechanisms, such as acetylation and methylation [124]. To date, no evidence has indicated that crotonylation occurs naturally on cysteine residues. The so-called “cysteine crotonylation” reported in the literature actually refers to an artificially designed strategy, in which a crotonyl-mimetic group (containing an α, β-unsaturated ester) undergoes an addition reaction with a proximal cysteine residue in the target protein, enabling covalent labeling and capture. This approach is primarily employed to enhance the interaction between probes and weakly binding proteins (e.g., Kcr-interacting proteins) and has been successfully applied to identify proteins such as HDAC1 and STAT3 [125].Crotonyl-CoA serves as a crotonyl group donor and contains an α, β-unsaturated double bond in the molecular structure. This structure increases the hydrophobicity of crotonylation and confers a unique spatial conformation, resulting in distinct modification sites and biological functions compared to those of acetylation. It should be noted that the occurrence of crotonylation involves two distinct mechanisms: in addition to the classical enzyme-catalyzed pathway, there exists a non-enzymatic process. Non-enzymatic crotonylation refers to the spontaneous chemical reaction in which the ε-amino group of a protein lysine residue directly reacts with the intracellular metabolite crotonyl-CoA, independent of specific transferase catalysis, thereby forming a crotonyl modification. This reaction is primarily driven by the chemical reactivity of the lysine side chain, and its frequency and extent are highly dependent on the intracellular concentration of crotonyl-CoA. Consequently, non-enzymatic crotonylation is regarded as a protein modification mechanism that directly and rapidly responds to changes in cellular metabolic state, working in concert with the canonical enzymatic modification pathway to constitute a dynamic and complex regulatory network for crotonylation [126]. For example, crotonylation plays a specific role in the structural dynamics of chromatin and regulates gene transcription [127].

Crotonylation is highly enriched in the active transcriptional regions of the genome, such as in gene promoters and enhancers, suggesting roles in activating transcriptional activation [128]. This modification participates in regulating gene expression and is closely linked to cellular metabolic states, playing vital roles in diverse physiological and pathological processes, such as metabolic reprogramming, maintaining stem cell pluripotency, immune responses, and tumorigenesis [129]. The equilibrium of crotonylation is primarily regulated through intracellular crotonyl-CoA levels, crotonyltransferase activity, and decrotonylase expression and function [130].

The biological effects of crotonylation are complex and context-dependent. For example, crotonylation exerts protective effects in acute kidney injury via upregulating PGC-1α and SIRT3 expression [131]. Crotonylation promotes macrophage activation and inflammatory cytokine release to regulate the immune system, alleviating neuropathic pain [132]. Crotonylation may promote invasion and metastasis in malignancies such as colorectal, lung, and pancreatic cancers [133]. Conversely, crotonylation potentially inhibits tumor progression via inducing cellular senescence and enhancing immune cell infiltration in hepatocellular carcinoma [134]. Furthermore, this modification may mediate immune evasion within tumor microenvironments such as glioblastoma through suppressing T-cell function and reshaping lysine metabolism to increase the tolerance of tumors to the immune system [134].

#### Enzymes regulating crotonylation

##### Crotonyltransferases

Crotonylation is catalyzed by specific crotonyltransferases that regulate downstream protein functions. The p300/CBP family proteins in mammals are the primary crotonyltransferases and can catalyze crotonylation at multiple sites on histones H3 and H4. This family was initially identified as classical HAT [127], and their crotonyltransferase activities were later discovered [135]. Their mechanism of action involves recognizing crotonyl-CoA as an acyl donor to transfer crotonyl groups onto the lysine residues of histones or nonhistones. In vivo crotonylation efficiency often relies on cofactors or conformational changes owing to the limited capacity of the p300/CBP catalytic pocket to accommodate long-chain acyl groups. The catalytic activity of p300 is regulated by multiple factors, such as p300 conformation, substrate concentration, and cofactor availability. The substrate specificity is influenced by the metabolic states of cells [136].Chemical proteom

Another class of crotonyl transferases is the PCAF/GCN5 family, whose members participate in regulating histone and nonhistone crotonylation. This family specifically modifies histone H3 at sites such as K9 and K14, participating in cell cycle regulation and DNA damage repair via altering the chromatin structure [137]. The affinity of the catalytic pockets of PCAF/GCN5 for medium-to-short acyl groups (e.g., crotonyl) is higher than that of p300/CBP. The catalytic pockets of PCAF/GCN5 maintain basal catalytic activity without the need for many cofactors. However, PCAF/GCN5 still requires association with proteins such as ADA2 and ADA3 to enhance modification efficiency and substrate specificity under specific conditions, such as high substrate concentrations or oxidative stress [138].ic approaches, such as photoaffinity probing, have been employed to directly capture and identify crotonylated substrates and interacting proteins of p300/CBP in complex cellular environments [139].

MYST family proteins, such as MOF and Tip60, possess crotonyltransferase activity, and help maintain chromatin homeostasis and cellular stress responses. The members of this family exhibit strong site-specific preferences for modifying histones. For example, MOF preferentially catalyzes crotonylation at H4K16, promoting an open chromatin conformation to support embryonic development and the expression of genes related to stem cell pluripotency [140]. Tip60 predominantly modifies H3K9 and H3K18 in the pathway that repairs breaks in double-stranded DNA [141]. The catalytic function of the MYST family is highly dependent upon the N-terminal zinc finger domain, in contrast to the first two enzyme classes. This region specifically recognizes crotonyl-CoA and synergistically interacts with chromatin remodeling complex components such as TRRAP to efficiently recognize and modify chromatin substrates without requiring cofactors [142]. The MYST family also acylates nonhistones, such as DNA polymerase η, contributing to the regulation of DNA replication fidelity. Key information regarding these crotonyltransferases is summarized in Table 5 to facilitate the comparison of the enzymes involved in regulating crotonylation. Notably, in vitro assays employing synthetic peptides may not fully recapitulate the physiological activity of enzymes on nucleosomal substrates within a chromatin context [41].

**Table 5:** Classification and names of enzymes associated with different acylation modification types

Modification Type	Enzyme Classification	Enzyme Name	Substrate	Experimental Types	References
Crotonylation modification	Crotonyltransferase	p300/CBP PCAF, GCN5, MOF	H3K18cr NPM1, DDX5 NPM1 DNA-PKcs DDX5	Nucleosome assay Synthetic peptide	[44, 47, 49] [143] [144] [145] [146] [147] [148]
	Decrotonylase	SIRT1, SIRT2, HDAC1, HDAC2	H1K26 H3K9, H3K56, H4K16 H3K9 H3K18, H4K16	Nucleosome assay Synthetic peptide	[69, 72, 73, 149] [150] [151] [152] [153] [154]
Malonylation modification	Malonyltransferase	MTₘᵢ	malonyl-CoA	Synthetic peptide	[59, 60, 104, 155]
	Demalonylase	Sirt3-S, SIRT5	Ago2, CPS1, GAPDH, P2, GLS		[156] [157] [158] [114] [159] [160]
Glutarylation modification	Glutaryltransferase	KAT2A, P300/ CBP	H4K91, H3, H4		[123, 135, 161] [162]
	Deglutarylase	SIRT5, SIRT7	CPS1, H4K91		[123, 138, 163] [161]

##### Decrotonylase

Decrotonylase is involved in maintaining the dynamic equilibrium of crotonylation by catalyzing the removal of the crotonyl groups from lysine residues on proteins. The Sirtuin family was the first group of enzymes identified as possessing histone decrotonylase activity. SIRT1 specifically targets histone H3K9 and H3K18 sites. The decrotonylase activity of SIRT1 aids in condensing chromatin, suppressing the expression of cell-cycle-related genes such as CCND1 and regulating cell proliferation as well as aging [164]. SIRT2 primarily localizes to the cytoplasm and mitochondria, where the decrotonylation of α-tubulin is catalyzed at position K40, regulating microtubule dynamics and influencing cell migration and mitosis [165].

Class I histone deacetylases (HDAC1/2) engage in histone decrotonylation in eukaryotes. HDAC1/2 form complexes with co-repressors, such as Sin3A. HDAC1/2 preferentially catalyze the decrotonylation of histones at positions H3K14 and H3K23, altering the structure of chromatin and inhibiting gene transcription. These enzymes also act on nonhistone substrates. For example, HDAC1 decrotonylases lysine 323 of the transcription factor c-Myc, reducing c-Myc stability and inhibiting tumor cell proliferation [166]. HDAC3 possesses decrotonylase activity and directly binds to DNA-PKcs to mediate DNA-PKcs decrotonylation. This weakens the ability of DNA-PKcs to bind to DNA and the Ku70/80 complex, inhibiting DNA-PK complex assembly and activation [147].

Decrotonylase activity is regulated by the intracellular metabolic cell environment. For example, short-chain fatty acids produced via gut microbiota metabolism, such as butyrate, inhibit HDAC activity, increasing histone acetylation [167]. Thus, nutritional status and metabolic products may influence cellular crotonylation by regulating decrotonylase activity. Key information regarding decrotonylase is summarized in Table 5 to facilitate a comparison of the enzymes regulated by histonylacetylation. Notably, in vitro assays employing synthetic peptides may not fully recapitulate the physiological activity of enzymes on nucleosomal substrates within a chromatin context [41].

#### Advances in chemical proteomics have propelled the functional characterization of protein crotonylation

Recent advances in chemical proteomics have significantly propelled the functional characterization of protein crotonylation. Global crotonylome profiling, enabled by high-resolution mass spectrometry, allows for the systematic identification and quantification of crotonylation sites within cells, revealing its extensive roles in transcriptional regulation and cellular metabolism [168]. For instance, a global crotonylomic analysis of cumulus cells during oocyte maturation not only mapped cell-type-specific modification landscapes but also identified critical non-histone substrates such as Annexin A2. The study further elucidated that Annexin A2, upon crotonylation catalyzed by EP300, activates the EGFR pathway to regulate cellular function, highlighting the power of comprehensive profiling in discovering functionally relevant modification targets [139].

Metabolic labeling techniques, which employ chemically tagged metabolic precursors, enable dynamic tracking and enrichment of crotonylation, offering spatiotemporally resolved tools to investigate its biological functions [169]. Open-search mass spectrometry strategies overcome the limitations of conventional database-dependent approaches, facilitating the discovery of novel modification forms and previously unrecognized interacting proteins, thereby expanding the understanding of crotonylation-mediated networks.

Furthermore, chemical probe-based methodologies provide powerful means to dissect specific interactions. For example, the developed "H3g27Cr" crotonyl-mimetic probe—wherein the crotonylamide moiety is replaced with a more reactive ester—enables specific covalent capture of proximal cysteine residues. This approach markedly enhances the identification of weakly interacting proteins such as STAT3, demonstrating the utility of chemical tools in elucidating the mechanisms of post-translational modification-mediated interactions [125]. Concurrently, the integration of photoaffinity labeling with genetic code expansion has led to the development of genetically encoded photo-crosslinking lysine crotonylation (Kcr) probes. These probes can be site-specifically incorporated into histone loci (e.g., H3K79) and, upon photoactivation, covalently capture "writer ", "eraser ", or "reader" proteins (e.g., the decrotonylase SIRT3) in living cells. This represents a significant breakthrough in achieving site-specific, in situ analysis of transient and weak interactions governed by crotonylation [170].

The integration and continued refinement of these diverse methodologies have collectively deepened our understanding of the biological functions of crotonylation and its implications in disease pathogenesis.

#### Crotonylation in tumor metabolism

Crotonylation participates in tumor metabolic reprogramming by affecting protein stability and localization.Crotonylation directly participates in processes such as glucose metabolism, DNA damage repair, and signaling pathway activation through regulating the stability and subcellular localization of tumor-associated proteins. Crotonylation thus drives tumor metabolic reprogramming.

Crotonylation modifies core tumor metabolic enzyme activity via regulating protein stability. The active states of key metabolic enzymes are engaged in reprogramming the metabolic processes of tumor cells. Crotonylation enhances the stability of these enzymes by altering protein conformation or interfering with the ubiquitin-dependent degradation pathway to produce metabolic abnormalities in the body.

PKM2 is a central regulator of the Warburg effect and directly influences the glycolytic rates in tumor cells through its protein stability. Crotonylation at lysine 498 (K498) of PKM2 inhibits PKM2 ubiquitination. This modification hinders the degradation of PKM2 via the proteasome pathway by blocking the binding between E3 ubiquitin ligases (e.g. CHIP) and PKM2, which increases intracellular PKM2 protein levels [171]. High PKM2 levels promote the accumulation of glycolytic intermediates in the dimeric form, providing the precursors for nucleic acid biosynthesis. Nucleic acid biosynthesis activates the transcription of oncogenes, such as MYC, through nuclear translocation, promoting tumor proliferation at the metabolic and transcriptional levels [172]. Conversely, SIRT1-mediated K498 decrotonylation enhances PKM2 ubiquitination and degradation, suppressing the protumor effects of PKM2. This demonstrates that crotonylation promotes glycolytic reprogramming by stabilizing PKM2 expression.

Nonhistone crotonylation modifications maintain metabolic stability in hepatocellular carcinoma cells. Zhang et al. [173] revealed the direct link between crotonylation modifications and the function of specific metabolic enzymes. Acyl-CoA oxidase 2 (Acox2)-knockout mice exhibited Acox2-deficiency-induced hepatocellular carcinoma. The mechanism underlying this effect involved the Acox2-specific downregulation of nonhistone crotonylation levels for multiple metabolic enzymes and peroxidases in liver tissue. This widespread reduction in crotonylation directly disrupted the normal function of these metabolic enzymes, severely impairing cellular metabolic homeostasis and inducing hepatocellular carcinoma [174]. Furthermore, crotonylation modulates the allosteric effects of key enzymes in glucose metabolism in hepatocellular carcinoma cells. This crotonylation reduces the production of ribose-5-phosphate and lactate, limiting the Warburg effect and inhibiting hepatocellular carcinoma cell growth.

Crotonylation modifications activate oncogenic signaling pathways by regulating subcellular localization of proteins..Crotonylation regulates oncogenic signaling pathways by influencing protein–protein interactions, protein stability, and subcellular localization [143, 173]. However, the regulation of protein function by crotonylation is highly environment-dependent. For example, multiple differentially expressed crotonoylated proteins localize to the cytoplasm and mitochondria in papillary thyroid carcinoma, where mitochondrial localization accounts for 17.5% of the proteins. Kcr may thus influence energy metabolism by regulating the localization of metabolism-related proteins. Functional enrichment analysis revealed the enrichment of these proteins in signaling pathways such as the PI3K-Akt, Hippo, and cell cycle pathways. Changes in the crotonylation levels of metabolic enzymes such as PKM and LDHA did not correlate with their protein expression levels, suggesting that Kcr abnormally activates oncogenic signaling pathways via modifying these proteins and altering their subcellular distribution to drive tumor progression [175, 176].

Crotonylation occurs at CD163 of phosphoglycerate dehydrogenase, located near the NADP⁺ binding pocket. Cryo-EM structural analysis revealed that crotonylation at Kcr induces a conformational change in the N-terminal domain of phosphoglycerate dehydrogenase, destabilizing the NADP^+^ binding pocket and inhibiting its enzymatic activity. Crotonylation at position K140 of transketolase and position K28 of aldolase C similarly inhibits their enzymatic activities via affecting the positioning of the catalytic core or intermolecular interactions [177]. These findings demonstrate the functional complexity of crotonylation, which inhibits the activity of certain oncogenic proteins and promotes gene transcription under specific conditions. The biological effect of O-GlcNAcylation depends on the specific protein modified and the cellular context.

### Malonylation

Malonylation is an evolutionarily conserved PTM of proteins that was discovered in mammals in 2011 [100]. Malonylation uses malonyl-CoA as a donor and is catalyzed by specific acyltransferases that covalently attach a malonyl group (-CO–CH₂-CO-) to the ε-amino group of the lysine residues in proteins [178]. SIRT5 is the primary deacylase and a central regulator of the dynamic equilibrium of malonylation [179]. Intracellular malonyl-CoA concentrations are regulated by acetyl-CoA carboxylase and malonyl-CoA decarboxylase, influencing the overall protein malonylation levels [102].

Malonylation introduces a net negative charge to the intrinsically positively charged lysine residue at physiological pH (7.2–7.4), neutralizing the original positive charge. This alteration markedly affects the electrostatic interactions between modified proteins and nucleic acids or other proteins. The spatial structure and electrochemical properties of malonyl groups induce localized changes in protein conformation, particularly at active enzyme sites or protein–protein interaction interfaces. These changes regulate target protein activity, stability, subcellular localization, and binding efficiency [180, 181]. Malonylation functions in metabolic diseases, such as diabetes and osteoarthritis, via regulating the activity of key enzymes involved in carbohydrate metabolism [182, 183]. Malonylation is also closely associated with tumor development and progression, such as in lung cancer and type 2 neurofibromatosis [184, 185].

Malonylation is widely distributed across diverse organisms, ranging from prokaryotes, such as *Escherichia coli* and *Saccharomyces cerevisiae,* to mammalian cells and tissues [186]. The subcellular localization reveals distinct compartmentalization of malonylation. Mitochondrial malonylation primarily targets and regulates the metabolic enzymes involved in fatty acid oxidation and the TCA. Cytoplasmic malonylation modifies key enzymes in the fatty acid synthesis pathway. Nucleic malonylation involves malonyl-CoA modification on histones and various non-histones present in regulatory regions, such as gene promoters and enhancers, suggesting direct involvement in the epigenetic regulation of gene transcription.

#### Enzymes regulating malonylase

##### Malonyltransferases

Malonyltransferase catalyzes the transfer of the malonyl groups on proteins from malonyl-CoA to lysine residues [186]. This enzyme participates in fatty acid synthesis and secondary metabolite modification and is primarily localized to the mitochondria [155].

Human mitochondrial malonyl-CoA transferase (MTₘᵢₜ) is a core component of the mitochondrial fatty acid synthase (FAS) II system and participates in de novo fatty acid synthesis within the mitochondria. This enzyme is encoded by a nuclear gene localized on human chromosome 22q13.31 and relies on the N-terminal mitochondrial targeting sequence for transport into the mitochondrial matrix. The absence of this sequence results in the cytoplasmic retention of the protein. Mature MTₘᵢₜ must be expressed and purified using the insect Sf9 cell system and functionally interacts exclusively with the mitochondrial acyl carrier protein (ACPₘᵢₜ, encoded by chromosome 16p12.3). Mature MTₘᵢₜ exhibits high substrate specificity, exclusively using malonyl-CoA as an acyl donor. MTₘᵢₜ forms a covalent malonyl–enzyme intermediate complex and specifically transfers the malonyl group to holo-ACPₘᵢₜ modified by human phosphopantetheine transferase [155]. The interaction between MTₘᵢₜ and ACPₘᵢₜ may supply long-chain fatty acid precursors for remodeling mitochondrial phospholipids or provide octanoyl-ACP for alpha-lipoic acid biosynthesis for maintaining mitochondrial respiratory chain function [155].

Certain HATs can use a wide range of substrates, catalyzing diverse acylation reactions such as malonylation. For example, the active site of p300/CBP contains a deep hydrophobic pocket that accommodates longer or charged acyl chains, providing the structural basis for catalyzing diverse acylation modifications. p300 catalyzes multiple modifications such as malonylation, succinylation, and glutarylation, in addition to acetylation [187]. Key information regarding malonyltransferases is summarized in Table 5, which describes the regulatory enzymes corresponding to malonylation modifications. Notably, in vitro assays employing synthetic peptides may not fully recapitulate the physiological activity of enzymes on nucleosomal substrates within a chromatin context [41].

##### Demalonylases

More demalonylases than malonyltransferases have been identified, with SIRT3 -S and SIRT5 being the most extensively studied. Sirt3-S is a cytoplasmic, NAD⁺-independent demalonylase subtype [188]. The core function of Sirt3-S is regulating Argonaute2 (AGO2) transport to the mitochondria via demalonylation. AGO2 exerts a protective effect in diabetic cardiomyopathy. SIRT3 -S expression is downregulated in diabetic cardiomyocytes under hyperglycemic conditions, leading to increased malonylation at lysine 440 (K440) of AGO2. This modification impairs the binding of AGO2 to the mitochondrial membrane transporter TIMM17B, inhibiting the translocation of AGO2 to the mitochondria. Sirt3-S restores AGO2–TIMM17B interactions by halting malonylation at K440, thus facilitating AGO2 mitochondrial translocation. AGO2 enters the mitochondria and further recruits the mitochondrial translation elongation factor TUFM, promoting the translation of CYTB. CYTB is a key subunit of the electron transport chain complex III. This process maintains the function of the complex to reduce the production of mitochondrial reactive oxygen species to ultimately mitigate high-glucose-induced cardiac dysfunction [189].

SIRT5 participates in the demalonylation process in addition to possessing desuccinylase activity. SIRT5 primarily localizes in the mitochondrial matrix, and its catalytic activity depends on the Tyr102 and Arg105 residues in the active site. These residues recognize and bind to the carboxyl group of malonyl-CoA through hydrogen bonds, ensuring substrate specificity [190]. SIRT5 extensively regulates mitochondrial protein deprocrystallization in various metabolic tissues, such as the brown adipose tissue, liver, and heart. SIRT5 is a core molecule that maintains energy metabolism and mitochondrial function [50]. For example, SIRT5 modulates the function of key metabolic proteins such as UCP1, glutamate dehydrogenase (GLUD1), and SDHA/B through decarboxylation in brown adipose tissue [191]. The SIRT5 targets in the liver and cardiomyocytes include glyceraldehyde-3-phosphate dehydrogenase and carbamoyl phosphate synthetase 1 (CPS1) [157]. The demalonylase activity of SIRT5 is also closely associated with tumor metabolism. SIRT5-mediated demalonylation of lysine 498 in PKM2 in lung cancer inhibits the nuclear translocation and glycolytic flux of PKM2, suppressing tumor proliferation [85]. SIRT5 maintains the antioxidant activity of isocitrate dehydrogenase 2 through demalonylation in hepatocellular carcinoma, reducing the reactive oxygen species (ROS) levels in the tumor microenvironment [6, 192].

These findings indicate that demalonylases play regulatory roles in metabolic diseases (e.g., diabetes and obesity) and tumors, serving as therapeutic targets. The regulatory enzymes corresponding to malonylation modifications are summarized with key information on demalonylases in Table 5. Notably, in vitro assays employing synthetic peptides may not fully recapitulate the physiological activity of enzymes on nucleosomal substrates within a chromatin context [41].

#### Malonylation in reprogramming metabolism in tumors

Malonylation uses malonyl-CoA as the core donor to support rapid tumor cell proliferation, oxidative stress adaptation, and invasive metastasis by regulating the reprogramming of glucose, amino acids, and lipids in cancer cells. Malonyl-CoA levels are regulated by key enzymes such as acetyl-CoA carboxylase (ACC). The subcellular distribution of malonyl-CoA and dynamic equilibrium of protein malonylation modifications collectively form the molecular basis for the metabolic dysregulation in tumors.

##### Malonylation in reprogramming glucose metabolism

Malonylation is more concentrated in the cytoplasm than is succinylation. Malonylation is involved in tumor metabolic reprogramming via directly regulating the activity of key glycolytic enzymes, such as PGK1 and PKM2 [85, 193]. PKM2 typically exists as a low-activity dimer in tumor cells. PKM2 promotes the accumulation of glycolytic intermediates that are diverted to the pentose phosphate pathway. Malonyl-CoA modification at lysine 498 (K498) of PKM2 causes substantially higher kinase activity than that of unmodified malonyl-CoA. This modification stabilizes the PKM2 tetrameric conformation, promoting pyruvate production and ATP synthesis and reducing dependence on the pentose phosphate pathway by decreasing NADPH generation. Tumor cell proliferation is ultimately inhibited. The deacetylase SIRT5 specifically removes the malonyl group at the K498 site, reverting PKM2 to a low-activity, dimeric state. This modification maintains the Warburg effect and enhances the ROS scavenging capacity of tumor cells by increasing the flux of the pentose phosphate pathway. These processes promote malignant growth in tumors such as lung and liver cancer [85]. Furthermore, malonylation at lysine 433 (K433) of PKM2 promotes translocation of PKM2 to the mitochondria under glucose deprivation. Malonylation at K433 stabilizes the mitochondrial membrane potential, reduces the release of mitochondrial apoptosis signals, and enhances tumor cell survival in nutrient-deprived environments by interacting with voltage-dependent anion channel protein 3 [194].

PGK1 is a key rate-limiting enzyme in glycolysis that catalyzes the conversion of 1, 3-bisphosphoglycerate to 3-phosphoglycerate while generating ATP. Changes in PGK1 activity directly impact glycolytic flux and cellular energy metabolism. PGK1 undergoes malonyl modification at lysine 131 (K131) during the differentiation of goat muscle preadipocytes. This modification specifically occurs on day 3 of differentiation [195]. The impact of PGK1 malonylation on the enzymatic activity of PGK1 has not been directly assessed; however, the time-specific modification at K131 suggests that glycolytic flux is regulated via the modulation of PGK1 catalytic function or protein interactions during a critical differentiation phase. This process provides energy and substrates for lipid deposition and regulates cellular metabolic reprogramming.

##### Malonylation in the reprogramming of amino acid metabolism

Glutamine is a nitrogen source for tumor cells that is catalyzed by GLS to form glutamate. Glutamine is oxidized by GLUD1 to α-ketoglutarate, which enters the TCA cycle for producing energy or participates in synthesizing glutathione [195]. The catalytic activity of GLUD1 is finely regulated by malonylation. SIRT5-mediated GLUD1 demalonylation enhances the catalytic activity of GLUD1, increasing the conversion of glutamate to α-ketoglutarate. This increased conversion promotes the flux of the TCA cycle to meet the synthetic and metabolic demands of tumor cells and simultaneously regulates serine hydroxymethyltransferase 1 (SHMT1) activity to promote the serine–glycine metabolic axis and one-carbon unit supply. These processes support nucleotide synthesis [196].

Mitochondrial transcription factor A deficiency in lung cancer cells induces metabolic reprogramming that elevates malonyl-CoA levels and triggers mDia2 malonylation. mDia2 malonylation promotes nuclear translocation, actin polymerization, and chromatin opening, ultimately upregulating metastasis-related gene expression and driving tumor metastasis [197]. SIRT5 deficiency in pancreatic cancer cells substantially increases malonyl-CoA modification levels across multiple key amino acid metabolism enzymes. Malonyl-CoA modification at the K160 site of glutamate-oxaloacetic transaminase 1 is prominent in this process. This modification inhibits the enzyme activity of glutamate-oxaloacetic transaminase, disrupts glutamine catabolism, reduces aspartate production, and impairs pyrimidine synthesis and reduces glutathione generation [198]. SIRT5 expression in lung cancer tissues positively correlates with GLUD1 activity, and patients with high SIRT5 expression exhibit larger tumors. This suggests that SIRT5-mediated demalonylation of GLUD1 is a key mechanism promoting energy metabolism and tumor growth in lung cancer [198].

In type 2 diabetes models, elevated levels of hepatic succinyl-CoA act as a direct donor of succinyl groups, which strongly promotes the succinylation of lysine residues in proteins. This modification particularly targets key enzymes in amino acid metabolism pathways, such as 10-formyltetrahydrofolate dehydrogenase, in one-carbon metabolism. The core mechanism involves modifying malonyl-CoA, which introduces negatively charged malonyl groups at conserved lysine sites within the active enzyme sites. This process involves electrostatic repulsion and steric hindrance, directly disrupting enzyme–substrate binding or altering the catalytic microenvironment. Enzyme activity is ultimately inhibited. Suppressing metabolic enzyme function accelerates systemic metabolic dysregulation, driving the pathological progression of diabetes [199].

##### Malonylation in reprogramming lipid metabolism

Lysine malonylation drives the reprogramming of lipid metabolism in tumor cells by regulating key enzymes and transcription factors involved in fatty acid synthesis, enhancing the invasion and metastatic capabilities of tumor cells. Malonyl-CoA, an acyl donor for this modification, is a metabolic intermediate in the fatty acid synthesis pathway that links protein function to cellular metabolic states, translating metabolic signals into protein-level regulatory events [200].

Fatty acid synthesis is primarily regulated at the molecular level by two rate-limiting enzymes: ACCα and FAS [201]. ACCα catalyzes the conversion of acetyl-CoA into acetyl-CoA, which serves as a substrate for FAS activity. Acetyl-CoA enters the FAS multienzyme complex together with acetyl-CoA and undergoes multiple reactions to synthesize palmitic acid. ACCα thus directly promotes FAS-mediated lipogenesis by regulating acetyl-CoA supply [202]. Malonyl-CoA also functions as a metabolic signaling molecule. Elevated malonyl-CoA levels inhibit CPT1 activity in the brain. Long-chain fatty acids are thus prevented from entering mitochondria for β oxidation [203], promoting the accumulation of fatty acids in the cytoplasm. These fatty acids are redirected toward synthesis pathways [204]. Elevated malonyl-CoA levels directly promote the synthesis of fatty acids, such as palmitic acid, for constructing tumor cell membranes and redirect lipid metabolism by inhibiting CPT1 to block fatty acid oxidation. Malonyl-CoA induces the lysyl malonylation of proteins, such as mTOR and glyceraldehyde-3-phosphate dehydrogenase, as a modification donor [118, 205], regulating metabolic enzyme activity and signaling pathways. The dynamic equilibrium of this process is maintained by the deacylase SIRT5.

SREBP-1 is a core transcription factor for synthesizing lipids that activates the expression of genes such as *ACCα* and *FAS* [206]. For example, SREBP-1 upregulation promotes *ACC1* expression in breast cancer, increasing malonyl-CoA production to accelerate fatty acid synthesis and support tumor growth. Obesity further enhances SREBP-1 activity and malonyl-CoA accumulation by activating the PI3K/AKT/mTOR pathway. This process increases lipid synthesis and drives breast cancer progression [207].

Tumor cells primarily rely on fatty acid synthesis [208]; however, the basal levels of fatty acid oxidation are crucial for maintaining energy under nutritional stress. Malonyl-CoA balances fatty acid synthesis and oxidation by competitively inhibiting CPT1 [209]. For example, malonyl-CoA inhibits CPT1 in cardiomyocytes to block fatty acid β oxidation, prompting cells to switch to glucose oxidation to obtain energy. This switch reduces the accumulation of toxic lipid intermediates during ischemia, increasing cardiac energy efficiency and protecting the myocardium from ischemia–reperfusion injury [210].

In summary, lysine malonyl-CoA modification profoundly influences lipid metabolic reprogramming in tumor cells by regulating fatty acid synthesis, oxidation, and key transcription factor activity. This modification directly promotes lipogenesis and supports oxidative function as well as coordinating glucose, amino acid, and lipid metabolic flux through malonyl-CoA-mediated metabolic inhibition and crosstalk signaling, enhancing tumor cell survival, proliferation, and invasiveness. Understanding the mechanisms involved in this modification mechanism can offer strategies for the development of targeted interventions.

### Glutarylation

Lysine glutarylation is a PTM that is chemically similar to succinylation and malonylation but possesses distinct functional and regulatory mechanisms [118]. This modification was first identified in mammalian cells by Tan et al. [163]. Glutarylation was subsequently observed to be widespread across organisms from bacteria to humans [211].

The unique properties of glutaryl modification primarily originate from the molecular structure of glutaryl. The glutaryl group possesses a hydrophobic chain of five carbon atoms with two terminal carboxyl groups, producing a strong double negative charge under physiological pH conditions [212]. This structural feature substantially alters the electrostatic properties and spatial conformation of proteins modified by glutarylation, influencing their activity, interactions, or stability [213].

Glutaryl-CoA is the primary acyl donor for glutarylation [214] and is a key metabolic intermediate in the pathways for degrading lysine, tryptophan, and hydroxylysine [215]. Glutaryl-CoA impairs mitochondrial TCA cycle function by inhibiting the E2 subunit of KGDHc [216]. This mechanism has been implicated in the pathogenesis of neurological disorders such as Alzheimer's and Parkinson's diseases [217, 218]. Furthermore, reduced glutarylation levels in the mitochondria of mammals may impair mitochondrial function, affecting spermatogenesis [217]. Similar to other lysine acylation modifications, glutarylation may play a role in cancer progression [163]; however, the specific mechanisms require further study.

#### Enzymes regulating glutarylation

##### Glutarylation transferases

Gultaryltransferases have been difficult to identify and classify functionally. Early studies suggested that high glutaryl-CoA concentrations chemically modify proteins through nonenzymatic mechanisms, similar to gultarylation [219]. However, multiple proteins exhibiting high pentanedioyl transferase activity have been identified with advancing researc [161, 220].

Certain acetyltransferases are compatible with a wide range of substrates. For example, KAT2A (GCN5) catalyzes not only histone acetylation and glutarylation but also the glutarylation of histones H3 and H4 [161]. The catalytic pocket of KAT2A is flexible, accommodating the long-chain dicarboxylic acid structure of glutaroyl-CoA. However, the catalytic efficiency of KAT2A for glutaroylation is lower than its activity for acetylation and succinylation [220].

P300/CBP is a broad-spectrum acyltransferase that primarily relies on its conserved HAT domain to recognize and bind acyl-CoA, such as glutaroyl-CoA. This binding transfers the acyl group to the ε-amino group of the lysine residues on target proteins. Although p300 can catalyze histone glutarylation in vitro, the physiological succinyl transferase function of p300 in vivo is not fully understood. p300 may share partial catalytic mechanisms with other acylation modifications (e.g., acetylation and succinylation), although substrate specificity and catalytic efficiency differ [163].

Histone glutarylation involves a unique enzymatic mechanism. Lysine acetyltransferase KAT2A forms a functional complex with the metabolic enzyme α-ketoglutarate dehydrogenase (α-KADH), coupling metabolic processes with epigenetic modification. In this process, α-KADH catalyzes the oxidative decarboxylation of α-ketoglutarate, directly generating glutaroyl-CoA. The adjacent KAT2A rapidly uses this product as a substrate, transferring the glutaroyl group to designated sites such as lysine 91 of histone H4 (H4K91) [161]. The regulatory enzymes corresponding to malonylation modifications are summarized in Table 5. Notably, in vitro assays employing synthetic peptides may not fully recapitulate the physiological activity of enzymes on nucleosomal substrates within a chromatin context [41].

##### Deglutarylase

SIRT5 is the most functionally characterized and catalytically efficient deglutarylase, with higher activity toward deglutarylation than desuccinylation [163]. SIRT5 contains an expanded acyl-binding pocket that specifically recognizes and accommodates long-chain dicarboxylic acid acyl groups. Arg105 and Tyr102 residues in the active site of SIRT5 stabilize the double-negatively charged terminal end of the malonyl group through electrostatic interactions, forming the structural basis for its highly efficient deglutarylation activity [163]. SIRT5 widely influences metabolic pathways such as the TCA cycle, amino acid metabolism, and fatty acid oxidation by regulating the glutarylation status of multiple mitochondrial enzymes, such as GDH, isocitrate dehydrogenase 2, and SCAD [221].

Unlike SIRT5, SIRT7 is primarily localized to the nucleus [222]. SIRT7 catalyzes deglutarylation at histone H3K18 in vitro and in cells, positioning SIRT7 as a deglutarylase. This catalytic process relies on NAD +, and the enzymatic activity of SIRT7 requires DNA involvement. SIRT7-mediated H3K18 deglutarylation is associated with chromatin condensation and transcriptional silencing. However, whether SIRT7 functions as a broad deglutarylation enzyme in vivo remains to be validated, whereas SIRT5 has been functionally well-defined. SIRT7 may operate as a complementary pathway under specific cellular environments or functional states [221, 223]. Key information regarding deglutarylase is summarized in Table 5, which describes the regulatory enzymes corresponding to glutarylation modifications. Notably, in vitro assays employing synthetic peptides may not fully recapitulate the physiological activity of enzymes on nucleosomal substrates within a chromatin context [41].

#### Glutarylation in reprogramming tumor metabolism

##### Glutarylation modifies tumor progression via regulating protein stability

Glutarylation modulates protein stability through multiple mechanisms, influencing tumor progression. For example, Verma et al. reported that glutarylation influences melanoma progression by regulating the stability of the transcription factor NRF2. Specifically, the loss of glutaryl-CoA dehydrogenase leads to intracellular accumulation of glutaryl-CoA, promoting NRF2 glutarylation. NRF2 glutarylation inhibits the binding of NRF2 to the E3 ubiquitin ligase KEAP1, reducing NRF2 ubiquitination and degradation, enhancing NRF2 stability, and DNA-binding activity [223].

The glutarylation modification of histone H4K91 is catalyzed by KAT2A using malonyl-CoA as a donor and can be reversed by SIRT7. This modification promotes transcriptional activation by reducing nucleosome stability and enhancing DNA accessibility, triggering cell cycle abnormalities, DNA repair defects, and telomere silencing dysfunction [224]. These alterations may contribute to genomic instability, indirectly promoting tumorigenesis and progression. However, whether glutarylation directly regulates nonhistone stability and the specific molecular mechanisms underlying glutarylation in tumors remain unknown.

The regulation of metabolic enzyme stability by glutarylation is substrate-specific and environment-dependent. For example, downregulated SIRT5 increases glutarylation levels of CPS1 under nutrient-deprived conditions. This process stabilizes CPS1 and increases urea cycle activity to supply essential intermediate metabolites to tumor cells [163]. Glutarylation markedly alters the electrophilic properties and spatial conformation of lysine residues, disrupting specific protein–protein interactions by introducing negatively charged glutaroyl groups. Glutaroylated GDH fails to bind key metabolic factors such as glutaroyl-CoA dehydrogenase. This disrupted interaction directly undermines the stability and function of mitochondrial metabolic enzyme complexes, reducing the efficiency of amino acid metabolism and the TCA cycle [211].

##### Glutaroylation regulation of subcellular protein localization

Glutaroylation affects protein stability and modulates the subcellular localization of key regulatory factors through controlling their interactions with molecular chaperones or the transport processes of nuclear pore complexes. Glutaroylation thus participates in regulating various cellular processes.

Glutaryl-CoA modification at multiple critical lysine sites (e.g., K889 and K1360) substantially inhibits enzyme activity and promotes nuclear accumulation as well as transcriptional regulation functions, using CPS1 as an example. This process involves multiple molecular mechanisms, such as allosteric regulation, site binding disruption, impaired oligomerization, and altered active site conformation. The deglutarylase SIRT5 specifically reverses this modification, restoring the normal function of CPS1 and maintaining ammonia metabolic homeostasis. This regulatory mechanism plays a central role in cellular metabolic adaptation, the energy balance, and the urea cycle, and accelerates tumor cell proliferation [163]. Furthermore, glutaryl-CoA modification enriched in outer-membrane proteins may promote the membrane localization of tumor-associated receptors or signaling molecules such as epidermal growth factor receptor (EGFR) or Wnt pathway components, activating downstream pro-proliferative pathways [225].

##### Glutarylation in reprogramming tumor metabolism

Glutarylation is a PTM primarily targeting metabolic enzymes, acting as a central regulator in reprogramming tumor metabolism. Glutarylation primarily occurs within the mitochondria, mainly because glutaryl-CoA predominantly localizes within the mitochondrial matrix. The higher pH in this region facilitates the deprotonation of lysine residues, promoting acylation reactions. The mitochondria are key organelles for energy production, cellular signaling, and survival. Therefore, mitochondrial dysfunction frequently leads to metabolic diseases and tumorigenesis. Glutaryl-CoA accumulation disrupts the mitochondrial energy metabolism by inhibiting the activity of the E2 subunit of KGDHc, affecting the TCA cycle and fatty acid oxidation processes. Glutaryl-CoA accumulation ultimately leads to TCA cycle dysfunction and energy metabolism disorders [214].

Glutaryl-CoA modification strongly inhibits the activity of multiple key metabolic enzymes. For example, GDH is an enzyme linking amino acid metabolism to the TCA cycle that catalyzes the oxidative deamination of glutamate to form α-ketoglutarate. Glutaryl-CoA glutarylation reduces enzymatic activity, causing glutamate accumulation and TCA cycle disruption, which forces tumor cells to increase glycolysis, promoting tumor growth and survival [211]. SIRT5-mediated deglutathionylation activates GDH, promoting glutamine breakdown and TCA cycle feedback reactions, and inhibiting SIRT5 considerably impairs the capacity of tumor cells to metabolize glutamine [226].

Glutarylation directly influences key metabolites such as glutaryl-CoA and α-KG by regulating glutaroyl-CoA dehydrogenase activity. α-KG is an essential cofactor for multiple dioxygenases, such as DNA and histone demethylases. Fluctuations in α-KG levels may indirectly affect the epigenetic states of cells. The accumulation of amino acids resulting from the inhibition of lysine oxidation activates nutrient-sensing pathways such as mTORC1 [215]. CPS1 is the rate-limiting enzyme in the urea cycle. CPS1 undergoes succinylation, which inhibits its activity, leading to ammonia accumulation and disrupting nitrogen homeostasis, further exacerbating metabolic abnormalities [163]. In summary, glutarylation plays a pivotal role in tumor progression by regulating metabolic and epigenetic processes.

## Acylation modifications on glycine

### Myristoylation

N-myristoylation is the most common form of acylation and an important PTM that is specific to eukaryotes [227]. N-myristoylation involves the attachment of a 14-carbon fatty acid myristate to the N-terminal glycine of proteins via N-myristoyltransferases. N-myristoylation thus affects physiological functions, such as the stability and cellular localization of proteins.

Proteins modified via N-myristoylation are more lipophilic, and N-myristoylated proteins are more often present in cells or Golgi membranes. Proteins with a hydrophobic region are more likely to localize to membranes after N-myristoylation. More than 100 N-myristoylated proteins were identified in a study published in *Nature Communications* in 2014. However, the authors did not validate the modifications of these proteins. Table 6 summarizes the effects of myristoylation on the subcellular localization or stability of proteins. Many proteins promote or suppress tumors after myristoylation [276, 277]. For example, two in vitro studies demonstrated that HPCAL1 obtained in vitro through chromatography and then modified with N-myristoylation readily adsorbed onto biofilms [278]. HPCAL1 acted as a substrate for NMT1 in another study, increasing the sensitivity of NMT1-high-expressing hepatocellular carcinoma cells to loratadine [279].

**Table 6:** Diseases affected by human myristoylated proteins and the pathways through which these proteins act

Protein	Disease	Localization	Stability	Year	Reference
ABL1	Chronic myeloid leukemia			2020	[228]
AIFM2	Ovarian cancer	√		2023	[229]
FMNL2	Ovarian cancer, breast carcinomas	√		2012; 2023	[230, 231]
LAMTOR1	Bladder cancer	√	√	2022	[232]
NCS1	Platinum-resistant ovarian cancer	√		2023	[229]
SCYL3	Breast cancer	√		2003	[233]
SRC	Prostate cancer	√	√	2017; 2010	[234–236]
AKAP12	Adipocyte	√		2022	[237]
ARF1	Diseases caused by aberrant STING activation	√		2023	[238]
ARF5	Cholera	√		1993	[239]
BASP1	Tumor suppressor activity			2022	[240]
CCNYCCNYL1	Psychiatric disorders	√		2022	[241]
DEGS1	Ceramide synthesis and lipoapoptosis	√	√	2012; 2009	[242]
HPCAL1;HPCA	Neuronal plasticity	√		2012	[243]
ZNRF2	Heart disease	√	√	2012	[244]
MSRA	Heart disease, lipid metabolism,	√		2011; 2018	[245, 246]
CAV2	diabetes			2020	[247]
ARF6		√		1995	[248]
ARL1		√		2004; 2001	[249]
CHCHD3		√		2012	[250]
CHMP6		√		2005	[251]
CHP1				2012	[252]
DDX46				2023	[253]
DMAC1				2023	[254]
FLOT2		√		2004	[255]
FMNL1		√		2009	[256]
FMNL3		√		2012	[230]
GNAI1		√		2017	[257]
GNAI3				2011	[258]
HCCS				2023	[254]
LNP		√		2013	[259]
LYN		√		2020	[260]
MARCKS		√		2000	[261, 262]
MARCKSL1		√		1998	[263]
MIC19				2019	[264]
MIC25				2018	[265]
NDUFB7		√		2023	[254]
PDE2A				2016	[266]
PLGRKT				2023	[254]
PPM1A		√		2013	[267]
PPM1B		√		2013	[267]
PRKAB1		√	√	2001	[268]
RFTN1		√		2003	[269]
RNF11		√		2010	[270]
RP2		√		2010	[271]
SVIP		√		2007	[272]
TESC			√	2023	[273]
TOMM40				2018	[265]
TXNRD1		√		2013	[274]
ZNRF2		√		2012	[275]

Lysine, in addition to glycine, can be myristoylated, and SIRT6 can reverse this process. SIRT6 is an NAD + -dependent deacylase that functions as a tumor suppressor. It is a lysine defatty-acylase that removes fatty acyl groups from R-Ras2, inhibiting the membrane localization of R-Ras2, activating the phosphoinositide 3-kinase (PI3K)/Akt pathway, and suppressing cell proliferation [275]. SIRT6 also removes the lysine fatty acylation modification from tumor necrosis factor-alpha (TNF-α), inhibiting the lysosomal targeting and degradation of TNF-α, which in turn promotes TNF-α secretion [280].

#### N-myristoylation involvement in tumor progression

N-myristoylation is involved in tumor progression by modifying proteins. N-myristoylation is an irreversible modification that is catalyzed by two N-myristoyltransferases, NMT1 and NMT2. NMT1 participates in tumor progression by influencing the chemical stability and physical hydrophobicity of proteins. NMT1 thus induces changes in protein expression and localization.

Inhibiting NMT1 activity inhibits the androgen-receptor-mediated progression of prostate cancer by augmenting the ubiquitin–proteasome degradation pathway and reducing androgen receptor levels. NMT1 affects the balance between N-myristoylation-downregulated and -upregulated proteins in liver cancer by influencing the activity of the ubiquitin E3 ligase HIST1H4H, which exacerbates liver cancer progression [281]. NMT1-driven myristoylation modifications occur on the EZH2 N-terminal motif G2 residue. The resulting hydrophobic interactions mediate liquid–liquid phase separation, forming multiple droplets in which STAT3 signaling is recruited and activated, contributing to lung cancer development [282].

Myristoylation affects the chemical stability and physical localization of proteins. For example, NMT1 inhibition inactivates the mTOR signaling pathway in bladder cancer by modulating the localization and stability of the late endosomal/lysosomal adaptor, MAPK, and mTOR activator 1 (LAMTOR1). SPI1 mediates NMT1 to promote the survival, migration, and invasion of gastric cancer cells by activating the PI3K/AKT/mTOR pathway. The NMT1-mediated myristoylation of CHP1 is the core mechanism through which the excessive membrane localization of programmed death ligand (PD-L1) induced by hypoxia and the subsequent immune evasion occur. Therefore, NMT1 can be targeted as a novel combination therapy to enhance the efficacy of immune checkpoint blockade [283].

NMT2 was first reported in 1998 [284]. It shares 77% homology with NMT1, but their functions do not completely overlap. NMT1 depletion primarily inhibits cell replication by suppressing proliferative signaling pathways, such as the c-Src and MAPK pathways. NMT2 loss more directly promotes apoptosis by altering the expression of BCL proteins [285]. NMT1 often promotes tumor cell proliferation during the progression of cancer, such as liver cancer. Studies targeting B lymphocytes have observed that NMT2 expression is selectively and substantially lower in hematologic cancers than in central nervous system, renal, and fibroblast-derived cancer cell lines [277]. This NMT2 expression leads to an increased dependence on NMT1, allowing for these cells to be selectively killed by inhibiting the remaining NMT1 activity. NMT2 expression serves as a survival marker for patients with acute myeloid leukemia (AML). Low NMT2 expression is associated with poor prognosis in these patients [286]. Immunohistochemical staining of malignant breast epithelial cells revealed that NMT2 was positively expressed in normal breast epithelial cells, whereas NMT2 expression was markedly lower in malignant breast tumors [280]. The findings of these two studies need to be further explored, and their specific underlying NMT2-related mechanisms require clarification.

NMT1 and NMT2 affect myristoylation to directly reduce the protein expression of these enzymes, which inhibits the myristoylation of downstream genes. For example, the myristoyl coenzyme A analog B13 inhibits NMT1 activity in prostate cancer. This inhibited NMT1 activity hinders the myristoylation of steroid receptor coactivator (SRC) proteins, which inhibits SRC protein phosphorylation, reducing the catalytic and scaffolding roles of SRC, and tumor progression is ultimately halted [287, 288]. However, using NMT1 or NMT2 as a direct tumor target requires further study. For example, NMT1 inhibited intracellular adhesion molecule 1 (ICAM-1) ubiquitin protease hydrolysis by preventing F-box protein 4 ubiquitin E3 ligase from interacting with ICAM-1, maintaining the adhesive capacity of tumor cells and inhibiting tumor metastasis. *NMT1* may function as an oncogene under certain circumstances. Thus, the comprehensive effects of NMT1 should be considered prior to using NMT1 as a therapeutic target [43, 46, 227, 232, 277, 280, 281, 285, 289–292] NMT1 may be involved in tumor progression by regulating its downstream signaling pathways [277].

### Palmitoylation

Braun and Radin demonstrated that the binding affinity of proteins to lipids is substantially weakened after palmitoylation through in vitro experiments in 1969 [293]. Proteins have been confirmed to undergo palmitoylation in vivo. The covalent attachment of 16-carbon palmitic acid to amino acid residues increases protein hydrophobicity; affects protein distribution, localization, and secretion; and regulates protein stability. Palmitic acid binds to proteins at different residues. Palmitoylation is classified as S-, O-, and -N-palmitoylation. S-palmitoylation involves the binding of palmitic acid to cysteine residues through thioester bonds. O- and N-palmitoylation involve the binding of palmitic acid to serine residues and amino termini of proteins, respectively. S-palmitoylation has been extensively studied, whereas O- and N-palmitoylation have only been reported in a few cases. For example, Wnt 3a is modified via O-palmitoylation, and hedgehog proteins are N-palmitoylated by hedgehog acyltransferase [294–296].

S-palmitoylation is mainly catalyzed by a family of proteins containing ZDHHC domains. The donor is usually palmitoyl coenzyme A. Two Zn^2+^ are coordinated by conserved cysteines and histidines, helping position the active site of the cysteine nucleophile, which is essential for catalyzing palmitoylation [297]. A total of 23 ZDHHCs have been identified in mammals. ZDHHC-catalyzed protein palmitoylation is a two-step process. The first step involves the self-acylation of cysteine in the active site of the ZDHHC enzyme. The second step involves the transfer of the palmitoyl group to the substrate cysteine by an acylase intermediate [298–300]. The deprotonated cysteine in ZDHHC nucleophilically attacks palmitoyl coenzyme A to generate an acylase intermediate [299]. The protonated His 154 likely donates a proton to the carbonyl oxygen in the second step, activating the acyl-cysteine intermediate. The enzyme-bound thioester is then used to transfer the palmitoyl group to the substrate [300]. The same type of ZDHHC is differentially expressed in different tumors. For example, ZDHHC2 expression is downregulated in pancreatic cancer and upregulated in renal cell carcinoma [301].

Depalmitoylation is catalyzed by palmitoyl protein thioesterase-1/2 (PPT-1/2), acyl protein thioesterase-1/2 (acylprotein thioesterase (APT)−1/2), and α/β hydrolase domain-containing protein 10/17 (ABHD 10/17). PPT-1 localizes to the lysosomes, depalmitoylates proteins prior to their degradation, and regulates the autophagy–lysosomal pathway [302]. PPT-2 similarly localizes to the lysosome and possesses a neutral optimum phase. However, PPT-2 is structurally different from PPT-1 and therefore utilizes a different catalytic substrate. The preferred substrate of PPT-2 is palmitoyl coenzyme A [303, 304]. APTase exhibits no substrate specificity and causes the generalized depalmitoylation of proteins on the intracellular membrane during the dynamic regulation of palmitoylation [305]. APT1 and APT2 possess overlapping functions but different actions in regulating palmitoylation dynamics. APT2 prefers to regulate polar proteins (e.g., Scribble) and membrane-associated enzymes (e.g., zDHHC6). APT1 plays a role in receptor signaling (e.g., β2-AR) and neuronal activity. Minor structural differences and subcellular localization determine substrate preference and inhibitor selectivity, suggesting that APT1 and APT2 exhibit different values as targets in disease interventions. The ABHD17 family contains depalmitoylated enzymes whose membrane localization depends on self-palmitoylation and form functional complements with APT enzymes [306]. Palmitoylation and depalmitoylation engage various physiological and pathological processes such as signaling pathways, cellular metabolism, tumor resistance, and immunosuppression. The following section focuses on the effect of palmitoylation on protein function, which has implications for tumor development and therapy.

#### Palmitoylation affects protein stability

Palmitoylation is a reversible long-chain lipid modification that affects the stability of numerous proteins and thus influences tumor progression. The palmitoylation of FASN mediated by ZDHHC21 decreases FASN protein stability and inhibits proliferation in diffuse large B-cell lymphoma cells. In contrast, palmitoylation increases oncoprotein stability and promotes tumor development in most cases. For example, the palmitoylation of EGFR is upregulated in metastatic colorectal cancer cells in the liver in nonalcoholic fatty liver disease, which promotes cell stemness by increasing EGFR protein stability and correcting plasma membrane localization. ZDHHC9 palmitoylates PD-L1 in pancreatic and lung adenocarcinoma, preventing PD-L1 degradation and maintaining PD-L1 stability to enable escape from antitumor immunity. The cysteine/glutamate reverse transporter SLC7A11 is a key regulator of iron resistance. ZDHHC8-mediated SLC7A11 palmitoylation in glioblastoma helps to maintain protein stability and resistance to iron-related death. Downregulation of the metabolic enzyme acyl-coenzyme A oxidase 1 (ACOX1) tumor suppressor increases β-catenin palmitoylation and stabilization and overactivates β-catenin signaling, promoting the progression of colorectal cancer [307–312]. Inhibiting protein palmitoylation in the tumor signaling pathway decreases protein stability, which blocks signaling and inhibits tumor cell proliferation, migration, and invasion. The decrease in FASN stability due to palmitoylation in diffuse large B-cell lymphoma may be related to the negative feedback regulation of lipid synthesis.

#### Palmitoylation affects protein location and distribution

Palmitoylation plays an important role in the membrane localization and distribution of proteins. ZDHHC13 is essential for classical tumor signaling pathways, EGFR sorting, and plasma membrane guidance [313]. ZDHHC-2 mediates S-palmitoylation of acylglycerol kinase (AGK) in renal clear-cell carcinoma cells, promoting the translocation of AGK to the plasma membrane [232] and activating the PI3K-AKT-mTOR signaling pathway. This process regulates the resistance of tumor cells to tyrosine kinase inhibitors. DHHC9 and DHHC15 are highly expressed in hypopharyngeal squamous cell carcinoma and assist in localizing Ras proteins to the membrane [314], promoting the malignant behavior of tumor cells. DHHC9 also assists in localizing the glucose transport protein, glucose transporter 1 (GLUT1), in gliomas [315]. Frizzled5 is a receptor in the classical Wnt signaling pathway. Palmitoylation is required for the transport of Frizzled5 from the endoplasmic reticulum to the Golgi apparatus, as well as for the further maturation of the Golgi apparatus [315].

The ZDHHC1-mediated palmitoylation of p53 is crucial for transporting p53 from the cytoplasm to the nucleus. Ras homolog family member U proteins are atypical members of the small G-protein family that bind to membranes via palmitoylation in prostate cancer [316], activating downstream p21-activated kinase. The protein limb expression 1 anchors to the outer mitochondrial membrane via palmitoylation to regulate the mitochondrial levels of ROS in gastrointestinal mesenchymal tumors [317]. Palmitoylation assists in the sorting, localization, and distribution of oncoproteins in the plasma membrane, promoting the malignant behavior of cancer cells. Depalmitoylation is a potential therapeutic strategy. Artemisinin may lead to the mislocalization of the small GTPase NRas in cancer cells by inhibiting ZDHHC6 activity, which disrupts the signaling cascade response of neuroblastoma RAS viral oncogene homolog (NRas) [318].

#### Palmitoylation participates in protein secretion

Palmitoylation participates in the subcellular localization and secretion of proteins into the extracellular compartment. Glycyl-tRNA synthetase 1 (GARS1) is a cytosolic enzyme that promotes apoptosis in cancer cells and is secreted by extracellular vesicles. Palmitoylation assists in the localization of GARS1 to extracellular vesicles and the secretion of GARS1 into the extracellular space [319]. Cytoskeleton-associated membrane protein 4 (CKAP4) is secreted from lung cancer cells through exosomes. Palmitoylation regulates the translocation of CKAP4 to the exosomes [320]. Thus, depalmitoylation inhibits oncoprotein secretion and affects tumor progression. For example, the inhibition of oncoprotein secretion mediated by WHN-88 effectively prevents the palmitoylation of Wnt ligands, a key PTM required for Wnt ligand maturation. This blocks their secretion and subsequent activation of the Wnt/β-catenin signaling pathway, which is frequently hyperactivated to drive tumor cell proliferation, invasion, and metastasis [321]. Mitogen-activated extracellular-signal-regulated kinase inhibitors in combination with PI3K inhibitors prevent the secretion of MUC2 by inhibiting MUC2 palmitoylation in mucinous colon/appendiceal cancer, promoting cancer cell apoptosis [322]. Palmitoylation may provide useful insights in developing targeted drugs. Toxic proteins were found to reversibly bind to extracellular vesicles via palmitoylation and kill cancer cells in coculture in vitro [323].

#### Palmitoylation affects protein structure

Protein structure is influenced by the number of carbon chains and inter-relationships between motifs. Protein palmitoylation alters the conformation and information transfer of proteins. Cluster of differentiation-44 protein (CD44) is a widely expressed cell membrane receptor that influences cell growth, migration, and tumor progression. CD44 dimerization is a key signaling event. Palmitoylation prevents the binding of CD44 to the cytoskeletal junctions of ezrin, radixin, and moesin and slightly alters the conformation of CD44, which is detrimental to CD44 dimerization [324, 325]. This process inhibits tumor progression and tumor cell metastasis. Gamma-secretase is an intramembrane protease that plays a critical role in Notch-dependent tumors. Palmitoylation of the gamma-secretase substrate alters the structure or increases the hydrophobicity of the transmembrane domain, preventing access to the active site of the enzyme. This, in turn, blocks Notch signaling and inhibits tumor progression [326].

#### Palmitoylation–effector protein interactions

**Fig. 3:**
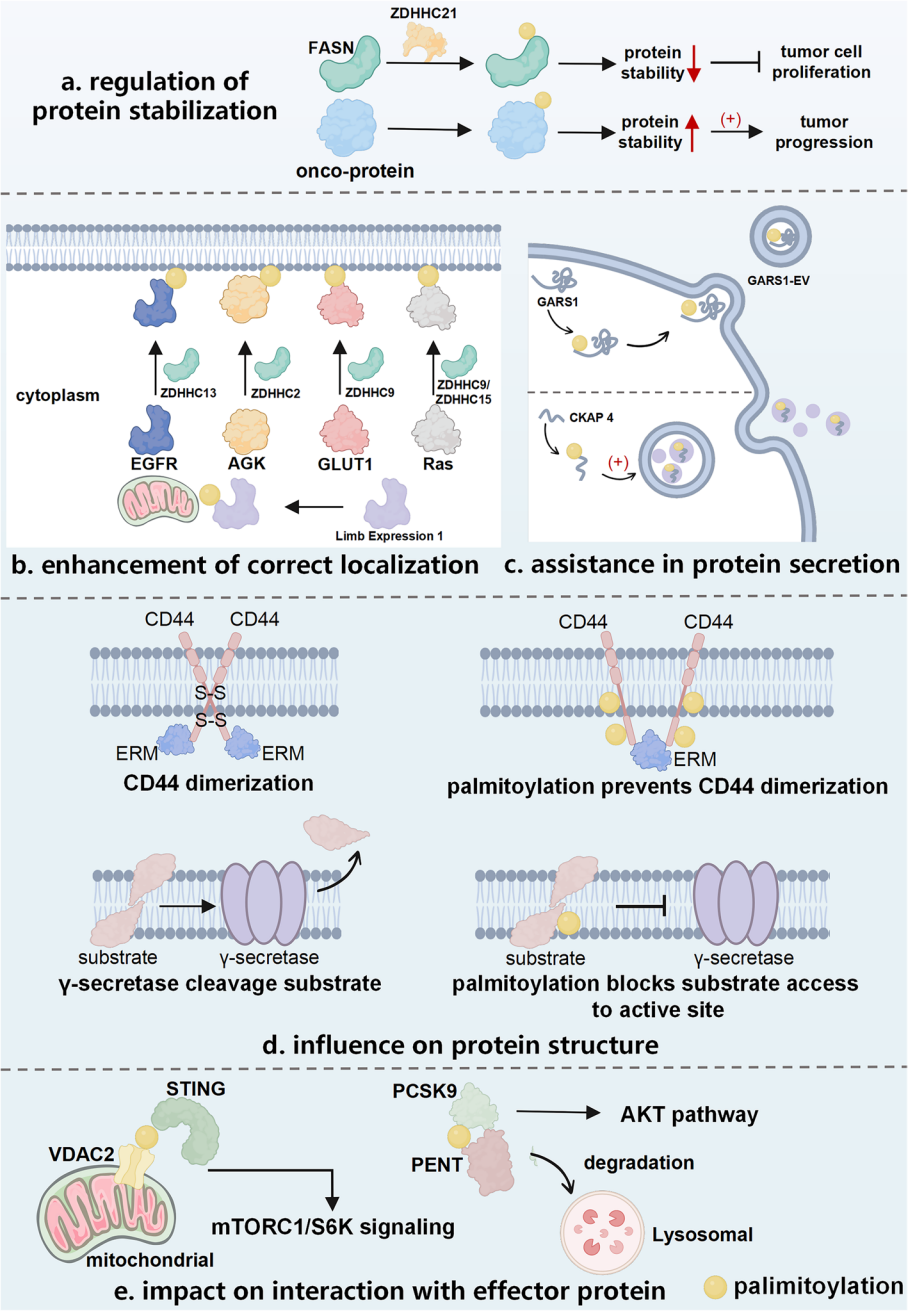
Palmitoylation affects various aspects of protein function. **a** Palmitoylation mediated by ZDHHC21 reduces FASN stability, inhibiting cancer cell proliferation. Palmitoylation of oncoproteins usually increases their stability, promoting tumor progression. **b** Subcellular localization: palmitoylation mediated by varying ZDHHCs assists EGFR, AGK, GLUT1, and Ras in binding to membranes. **c** Palmitoylation assists with extracellularly secreting GARS1 and CKAP4 via extracellular vesicles and exosomes, respectively [322]. **d** Palmitoylation prevents CD44 dimerization and blocks substrate access to the active site [324]. **e** Palmitoylation facilitates the interaction of stimulator of interferon genes with the effector protein VDAC2, thereby activating mTORC1/S6K signaling. Palmitoylation also enhances the interactions between PCSK9 and PTEN, promoting the lysosomal degradation of PTEN and subsequently activating the AKT pathway [330]

Interactions between proteins are generally mediated by specific structures, and palmitoylation acts as a motif in protein interactions. The innate-immune-associated factor stimulator of interferon genes interacts with the mitochondrial voltage-dependent anion channel 2 (VDAC2) via palmitoylation to activate mTORC 1/S6K signaling and promote renal carcinoma cell growth [327]. The palmitoylation of protein convertase subtilisin/kexin type 9 (PCSK9) enhances the interaction between PCSK9 and tensin homolog (PTEN) [328], causing PTEN to undergo lysosomal degradation and activate the AKT pathway. This process is involved in the development of sorafenib resistance in hepatocellular carcinoma. Specific occupation of the palmitoylation site inhibits interactions with effector proteins, blocking the signaling pathway and inhibiting tumor progression. For example, mCMY020 occupies the palmitoylation site of TEA region transcription factors (Teads) and inhibits the binding of Yes-related proteins to Teads [329]. In summary, palmitoylation affects tumor progression by influencing protein stability; membrane localization and sorting; secretion; structure; and interactions (Fig. 3).

## Targeted delivery systems for acylation modifications and clinical trials

This section summarizes the origins and functions of the donors for the four protein acylation modifications. We highlight the research progress on inhibitors targeting these four proteolipid acylation modifications for treating cancer, including those in Phase I clinical studies and those that have been commercialized. We also summarize the development of targeted delivery systems for some of these inhibitors.

### Acylation donors, writers, erasers, and readers

Protein acylation is governed by nonenzymatic and enzymatic regulatory processes, the latter of which is most prevalent. Acylation is typically controlled through a dynamic equilibrium between acyltransferases (writers) and deacetylases (erasers). Writers function through attaching acyl groups from acyl-CoA donors, such as acetyl, succinyl, myristoyl, and palmitoyl groups, to the side chains of lysine (or other) residues. Erasers catalyze the stripping of acyl groups from the amino acid residues. Specific protein structural domains (readers) then identify these acylation modifications (Table 7).

**Table 7:** Donors, writers, erasers, and readers of four types of cysteine acylation

Acylation	Donor	Writer	Eraser	Reader	
Myristoylation	Myristoyl-CoA: edible oil (coconut oil, butter, and palm oil)	NMT family (NMT1 and NMT2)	SIRT6, IpaJ	NA	
Palmitoylation	Palmitoyl-CoA: edible oil (coconut oil, butter, and palm oil)	ZDHHC family (ZDHHC1–23)	APT, PPT, ABHD	NA	[331]

#### Protein acylation donors

**Fig. 4:**
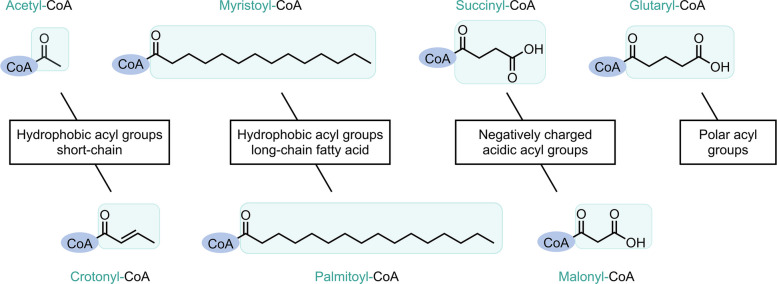
Chemical structures of seven acyl-CoA types. Based on the chemical structure of the acyl-CoA, the seven acyl groups can be classified into three categories: hydrophobic short-chain acyl groups (acetyl and crotonyl), long-chain hydrophobic fatty acyl groups (myristoyl and palmitoyl), and negatively charged acidic acyl groups (succinyl and malonyl). Glutaryl is a polar acyl group

Donors provide acyl groups required for protein acylation. The seven acyl groups are classified into three categories based on the acyl-CoA structure: hydrophobic short-chain acyl groups (acetyl and crotonyl); long-chain hydrophobic fatty acyl groups (myristoyl and palmitoyl); and negatively charged acidic acyl groups (succinyl and malonyl), whereas glutaryl represents a polar acyl group (Fig. 4). The different chemical acyl-CoA structures produce varying effects on the physicochemical properties of the substrate proteins, such as the hydrophobicity and charge. Acyl-CoAs typically serve as primary acyl donors and are predominantly sourced from the metabolites of glucose, fatty acids, and amino acids, via the major metabolic pathways in the human body. Exploring their sources has broadened the scope of protein acylation studies, as detailed in the following sections.

#### Donors for protein acetylation

Acetyl-CoA serves as a donor for protein acetylation; it is abundant in cells and is a crucial metabolite and substrate in numerous metabolic processes. Mitochondrial acetyl-CoA is produced through glycolysis [332], lipid β-oxidation [333], and the breakdown of branched-chain amino acids [334]. Acetyl-CoA is generated in the cytoplasm via the ATP-dependent reductive carboxylation of glutamine and acetate, after which it diffuses freely into the nucleus. Two nuclear enzymes (ATP-citrate lyase and acetyl-CoA synthetase) generate acetyl-CoA [335–337]. Furthermore, acetyl-CoA can be produced through specific metabolic pathways, such as β-hydroxybutyrate in neurons and the ethanol–acetaldehyde–acetic acid axis in hepatocytes or brain cells [338, 339].

#### Donors for other protein acylations

Protein acylation has been less studied than protein acetylation. The three main succinyl-CoA sources are (1) short-chain fatty acids and carboxylic acids, such as succinate and α-KG with succinyl-CoA primarily generated from α-KG through α-KGDHC in the TCA cycle [89], (2) amino acid metabolism in which isoleucine, methionine, and valine are metabolized into intermediates that are further converted into succinyl-CoA before entering the TCA cycle, and (3) other acyl-CoA types, such as methylmalonic acid, which is isomerized into succinyl-CoA by methylmalonyl-CoA mutase, a vitamin-B12-dependent enzyme [340]. Long-chain fatty acid acyl-CoAs, such as myristoyl- and palmitoyl-CoA, are typically synthesized from naturally occurring long-chain fatty acids present in edible oils, such as coconut oil, butter, and palm oil.

#### Writers of protein acylation

Writers are enzymes that mediate covalent modifications of particular residues, including KATs, NAT, DHHC, and NMT families.

##### Writers of protein acetylation

KATs are enzymes responsible for catalyzing lysine acetylation [341]. KATs consist of different families of NATs, including the GCN5-related family (GNAT), represented by GCN5 and PCAF; the p300/CBP family, consisting of p300 and CBP; MOZ, MOF, Ybf2 (Sas3), Sas2, TAT-interacting protein 60 (Tip60), and monocytic leukemia zinc finger proteins; and the SRC family, represented by SRC1–3. Additionally, some acetyltransferases, such as acetyl-CoA acetyltransferase 1 (ACAT1) do not belong to this category [46].

##### Writers for protein succinylation

KATs exhibit strong acyltransferase activity and catalyze a variety of acylation reactions. For example, succinylation can be catalyzed by p300/CBP and GCN5 in KATs. CPT1A functions as a specific writer of Ksus in addition to KATs [75].

##### Writers of protein myristoylation and palmitoylation

NMT is the main enzyme responsible for myristoylation [342]. Higher eukaryotes use two major NMT isozymes: NMT1 and NMT2 [285]. Protein palmitoylation can be classified into three types depending on the palmitoylation site: N-, O-, and S-palmitoylation [343]. The palmitoyl group in protein palmitoylation is linked to the target protein via PAT, which possesses the DHHC motif [344]. DHHC PATs belong to the ZDHHC family, which includes 23 distinct members in mammals. Buglino and Resh [345] developed a method for detecting palmitoylated modifications in sonic hedgehog (Shh) proteins. This technique permits highly efficient purification of hedgehog acyltransferase (Hhat) and provides biochemical data. These data directly indicate that Hhat functions as a PAT for Shh proteins. Specifically, Hhat exploits its structural features to attach a palmitoyl group (from palmitoyl-CoA) to the N-terminal cysteine residue of Shh proteins, forming a thioester linkage. These findings reveal a functional mechanism and provide a fresh perspective on protein palmitoylation.

Rana et al. [300] described the structures of a human DHHC enzyme designated DHHC20 and a zebrafish version of another human DHHC enzyme designated DHHC15. Blocking DHHC20 renders cancer cells more sensitive to Food and Drug Administration-approved drugs targeting EGFR.

#### Erasers of protein acylation

Erasers mainly belong to the HDAC and APT families. The function of erasers is the opposite to that of writers, in that they remove protein PTMs. HDACs are erasers of protein acetylation, removing acetyl groups from lysine residues. HDACs comprise two main families: the NAD^+^-dependent sirtuin family (SIRT1–7) and Zn^2+^-dependent HDAC family (HDAC1–10) [187]. Succinylation and acetylation erasers overlap and they exhibit high desuccinylase activity but low deacetylase activity [346]. SIRT7 is also a histone desuccinylase that plays a role in the response to DNA damage [183], although SIRT7 is not highly active [34].

Eraser of protein myristoylation and palmitoylation.

SIRT6 primarily mediates the demyristoylation of lysine residues. The unique structure is characterized by a large hydrophobic pocket, enabling the removal of myristoyl groups [347]. Additionally, plasmid antigen J (IpaJ), a myristoylase for human ARF1, was identified by Burnaevskiy et al. [348] as a type III effector from *Shigella flexneri* that exhibits cysteine protease activity. Depalmitoylation is catalyzed by members of the almitoyl-protein thioesterases superfamily, such as acyl protein thioesterases (APT1/2), palmitoyl protein thioesterases (PPT1/2), and α/β hydrolase-domain-containing protein 17A/B/C (ABHD17A/B/C).

#### Readers of protein acylation

Readers are proteins that specifically recognize modification sites on other proteins. They typically contain a conserved structural domain that is identified as a writer or an eraser, aiding in catalytic processes. Common examples of readers are bromodomains, YEATS (Yaf9, ENL, AF9, Taf14, and Sas5), and plant homologous domain (PHD) finger proteins. Only bromodomain types (BRD4, BRD3, and PBRM1) recognize acetylated nonhistone proteins. Bromodomain types are all transcription factors. BRD4 and BRD3 use different bromodomain structural domains (BD1, BD2, or both) to identify acetylated nonhistone proteins that subsequently regulate their transcriptional activity or protein degradation [46]. However, the selectivity and mode of operation of these structural domains remain unknown and thus require further investigation. The bromodomain is a classic reader of histone acetylation. However, the bromodomain also binds to other acylations, such as Kpr and Kbu [294]. YEATS and PHD are readers of the Kac histone, but they possess a higher affinity for acylation than acetylation [349–355].

Most research investigating protein acylation readers has focused on Kac and Ksuc histones as well as nonhistone Kac, and relatively little attention has been paid to the readers of long-chain fatty acid lipidation of histone and non-histone proteins. Further studies are required to identify the potentially unknown readers.

### Acylation inhibitors and targeted delivery systems

Metabolomic and proteomic technologies have increased our understanding of the roles of protein acylation in the pathogenesis of various diseases, particularly cancer. The four acylation modifications we have discussed regulate tumor biological behaviors through various molecular mechanisms. Acetylation targets the activation of the pro-oncogenic signaling pathway by enhancing the ability of SMAD3 to interact with the oncogenic chromatin-modifying protein TRIM24. Acetylation blocks the binding of SMAD3 to the oncogenic protein TRIM33. Myristoylation regulates the subcellular localization and activity of proteins by altering the hydrophobicity and chemical stability of these proteins. Succinylation targets metabolic enzymes and systematically reconfigures the networks for metabolizing glucose, lipids, and amino acids. Palmitoylation affects the malignant phenotypes of tumor cells by dynamically regulating the stability, transmembrane localization, and interaction networks of membrane proteins. All four modification types rely on specific enzyme systems to reversibly regulate function, but their targets differ. Acetylation regulation affects transcription factor interactions, succinylation mainly impacts metabolic reprogramming, myristoylation mainly changes the physicochemical properties of proteins, and palmitoylation integrates the functional modules of membranes. However, all modification types promote tumor cell phenotypic plasticity through covalent modifications, highlighting the synergistic roles of PTMs in regulating tumor heterogeneity.

These findings reveal the deep integration of metabolic reprogramming and signal transduction, providing a theoretical basis for the development of specific antitumor strategies targeting acyltransferases or deacylation enzymes. Published studies will facilitate the clinical translation of acylation-based precision therapy. Appropriate measures, such as the use of inhibitors or activation of regulators that influence the acetylation state of proteins, can be identified as the regulators and their influence on protein acetylation (e.g., writers, erasers, and starvation) are identified. These measures could further affect the upstream and downstream functions of the regulators, or their interaction with other biochemical processes, regulating disease progression.

The main enzymes currently being investigated are inhibitors of the various enzymes required for acylation modifications. Therefore, we briefly describe the research progress on the inhibitors associated with the four protein acylation modifications. We cover those in Phase I clinical studies and those that are commercially available.

### Inhibitors and cases

#### Acetylation-related inhibitors

The primary KAT constituents belong to the p300/CBP family of proteins, which is crucial for regulating numerous cellular processes such as the cell cycle, proliferation, and differentiation. These p300/CBP proteins are frequently overexpressed in multiple cancer types such as colorectal, breast, liver, and prostate cancers, as well as in several forms of leukemia [356–360] Pharmacological agents targeting p300/CBP have been extensively investigated. Over 135 compounds activate or inhibit p300/CBP and are classified into two categories based on the structural domains of the target proteins: either HAT or BRD domain [361].

CCS1477 is an orally bioavailable, potent, and selective inhibitor of the p300/CBP bromodomain. CCS1477 inhibits prostate cancer cell propagation and reduces the expression of genes regulated by the androgen receptor (AR) and cellular Myc [358]. The inhibitor FT-7051 was designed to bind strongly and specifically to the p300/CBP bromodomain, effectively blocking androgen binding and reducing AR activation. The safety and efficacy profiles of FT-7051 for treating metastatic castration-resistant prostate cancer were favorable in Phase I clinical trials. P31670 (NEO2734) is an innovative and highly efficacious oral drug that inhibits bromodomain extraterminal (BET) and p300/CBP. Dual inhibitors exhibit a stronger capacity to eradicate tumors across a wide range of solid tumor types than do nondual BET inhibitors [362]. C646 and A485 are among the compounds targeting the HAT structural domain. These compounds are in the early stages of development, but they exhibit highly selective antitumor effects.

Cellular HDAC regulation is closely associated with cancer progression. Therefore, targeting HDACs is a promising strategy for cancer therapy. Seven HDAC inhibitors have been globally approved and marketed: vorinostat, panobinostat, belinostat, romidepsin, chidamide, givinostat, and entinostat (Table 8). Vorinostat, belinostat, and panobinostat are pan-HDAC inhibitors, whereas chidamide is a selective HDAC1–3 and HDAC10 inhibitor. However, the role of romidepsin as a selective HDAC or pan-HDAC inhibitor is not fully understood [46, 363]. Givinostat and entinostat were approved in 2024 and have exhibited efficacy in clinical trials for the treatment of Duchenne muscular dystrophy and breast cancer, respectively [364, 365].

**Table 8:** Seven commercially available HDAC inhibitors and their outcomes

Compound	Target	Mechanism	Indications	Outcomes	Patent Publication No
Vorinostat	Pan HDACs	Inhibits accumulation of acetylated histone and nonhistone proteins	Cutaneous T-cell lymphoma	Combined therapy more effective than single therapy	WO1993007148A1
Romidepsin	Indeterminate	Inhibits HDAC1/2 activity, promoting cell apoptosis	Cutaneous T-cell lymphoma	Activity of two combination therapies was high with acceptable safety profile but did not increase CHOP efficacy in PTCL	EP0352646A2 WO2006129105A1
Belinostat	Pan HDACs	Inhibits accumulation of acetylated histone and nonhistone proteins, inducing autophagy	Cutaneous T-cell lymphoma	Combination therapy had limited activity but was highly toxic	WO2002030879A2
Panobinostat	Pan HDACs	Inhibits accumulation of acetylated histones and nonhistone proteins, promoting autophagy and apoptosis	Cutaneous T-cell lymphoma	Efficiency of most combined therapies was higher than that of single therapy	WO2002022577A2
Chidamide (tucidinostat)	Class I HDACs and HDAC10	Induces acetylation of H3 proteins	Peripheral T-cell lymphoma	Combined therapy was safe and valid in non-Hodgkin’s lymphoma	WO2004071400A2
Givinostat	Pan HDACs	Alleviates muscle tissue damage and protects muscle microfibers via inhibiting abnormally elevated HDAC activity	Duchenne muscular dystrophy and polycythemia vera	Approval based on results of multinational Phase III EPIDYS trial: givinostat recipients declined less than placebo recipients in time to perform a functional task	WO1997043251A1
Entinostat	Class I HDACs	Inhibits cell proliferation and promotes apoptosis in breast cancer	Breast cancer	Entinostat plus exemestane significantly increased progression-free survival compared to exemestane alone in patients with HR +/HER2– ABC that progressed after endocrine therapy	EP0847992A1

Although HDAC inhibitors have demonstrated antitumor effects against leukemia as monotherapies, or even against some neuromuscular diseases, their efficacy against solid tumors is low. Consequently, clinical trials are increasingly examining the potential of combining HDAC inhibitors with other anticancer agents for treating a wide range of tumors [366]. The efficacy of commercialized HDAC inhibitors in combination with other anticancer agents was higher than that of monotherapies in several clinical trials [367]. The findings of a large-scale, randomized Phase III study indicated that increasing the cytarabine dose did not result in superior therapeutic outcomes during induction therapy in young patients with AML, regardless of the concomitant use of vorinostat [368].

Numerous novel inhibitors are currently undergoing clinical trials. For example, the pan-HDAC inhibitor CI-994 has progressed to Phase II and III trials for treating lung cancer, multiple myeloma, and pancreatic cancer. According to a Phase II randomized, double-blind, placebo-controlled multicenter study, the combination of gemcitabine and CI-994 was not more efficacious than gemcitabine alone in patients with advanced pancreatic cancer [369]. A Phase 1a clinical trial investigated the histone deacetylase inhibitor bisthianostat, an HDAC inhibitors drug with a safety profile in the therapeutic management of relapsed/refractory multiple myeloma, for treating multiple myeloma. Bisthianostat exhibits favorable pharmacodynamics, pharmacokinetics, and therapeutic efficacy [370]. Furthermore, numerous additional drugs are undergoing Phase I and II clinical trials for treating solid tumors or leukemia (Table 9).

**Table 9:** Drugs targeting HDACs that have entered clinical trials for treating cancer

Drug	Target	Mechanism	Indications	Clinical stage	Clinical trial IDs or patent number
Abexinostat	Pan HDACs	Higher selectivity of HDAC6/8	Renal cell carcinoma	Ⅲ	NCT03592472
CI-994	Pan HDACs	Inhibits activity of HDAC1, 2, 3, 8	Pancreatic cancer, multiple myeloma, pancreatic cancer	Ⅱ	NCT00005624 NCT00005093 NCT00004861
Domatinostat (4SC-202)	Class I HDACs	Induces hyperacetylation of H3 histones	Merkel cell carcinoma, GI cancer	Ⅱ	NCT04393753 NCT03812796
Tinostamustine (EDO-S101)	Class I, II HDACs	Inhibits HDAC activity to promote histone and nonhistone acetylation	Numerous solid tumors	Ⅰ/Ⅱ	NCT03687125
KA2507	HDAC6	Selectively inhibits HDAC6 to regulate histone and nonhistone acetylation levels	Solid tumor,	Ⅰ	NCT03008018
CXD101/ Zabadinostat	Class I HDACs	Inhibits activities of HDAC1, 2, and 3	Advanced cancer	Ⅰ/Ⅱ	NCT01977638 WO2012120262A1
Tasquinimod	HDAC4	Inhibits HDAC4 signaling through mutagenesis	Multiple myeloma	Ⅰ	NCT04405167
AR-42	Pan HDACs	Inhibits HDAC activity, affecting multiple signaling pathways, such as Akt	Recurrent plasma cell myeloma, adult acute myeloid leukemia, Vestibular Schwannoma, meningioma	Ⅰ	NCT02569320 NCT01798901 NCT02282917
Citarinostat	HDAC6	Inhibits HDAC6 activity to increase acetylation level of certain nonhistone substrates	Smoldering multiple myeloma	I	NCT02886065
Bisthianostat	Pan HDACs	Inhibits HDAC activities to prevent deacetylation of histones, leading to accumulation of highly acetylated histones	Multiple myeloma	Ⅰ	NCT03618602
Purinostat mesylate	class I and IIb HDACs	Selectively inhibitsHDAC inhibitors and IIb, inducing apoptosis; downregulates BCR-ABL and c-MYC expression in Ph leukemia cell lines and primary Ph B-ALL cells from relapsed patients	BCR-ABL-induced B-cell acute lymphoblastic leukemia	Ⅱ	WO2022110568A1
Resminostat	class I, IIb, and IV HDACs	Induces high histone H4 acetylation and apoptosis in MM cells	Hodgkin's lymphoma, solid tumor	Ⅱ	WO2005087724A2
Tefinostat	Pan HDACs	Induces apoptosis and inhibits growth	Chronic granulomonocytic leukemia; hepatocellular carcinoma	Ⅰ	NCT02759601 NCT00820508
KA-2507	HDAC6	Inhibits HDAC6, restoring cell ciliation and attenuating malignant phenotype	Solid tumors	Ⅰ	NCT03008018
Pracinostat	Pan HDACs	Increases expression of cell cycle protein-dependent kinase 5 and induces acetylation	Locally advanced or metastatic solid tumors, hematologic malignancies,	Ⅰ	NCT00504296 NCT00741234 NCT01075308
OKI-179	Class I HDAC	Induces apoptosis and increases histone acetylation	Advanced solid tumor	Ⅰ/Ⅱ	NCT03931681 NCT05340621
Trichostatin A	Pan HDACs	Inhibits HDACs, may increase histone acetylation level, promoting activity of genotoxic anticancer drugs such as cisplatin via increasing DNA accessibility	Relapsed or refractory hematologic malignancies	Ⅰ	NCT03838926
JBI-802					
Ivaltinostat	Pan HDACs	Induces cell death via modulating p53 acetylation; induces antitumor effects via miRNAs targeting the Hippo pathway in cancer cells	Pancreatic adenocarcinoma	Ⅰ/Ⅱ	NCT05249101
MPT-0E028	Pan HDACs	Inhibits tumor cell proliferation and promotes tumor cell apoptosis	Advanced solid malignancies	Ⅰ	NCT02350868

Bromodomain and BET inhibitors are beneficial in cancer treatment. More than 50 BRD3/BRD4 inhibitors have been developed, including PLX51107, OTX015/birabresib, and BI 894999, most of which have entered clinical trials (Table 10).

**Table 10:** Drugs targeting BRD3/BRD4 that have entered clinical trials for treating cancer

Drug	Target	Mechanism	Clinical stage	Indications	Clinical trial IDs
PLX51107	BRD3, BRD4	Blocks some enzymes needed for cell growth	Phase I	Acute myeloid leukemia	NCT04022785 NCT02683395
OTX015	BRD3, BRD4	Blocks interaction of BRD3 and BRD4 with acetylation markers (e.g., AcH4) on chromatin	Phase I	Acute myeloid leukemia	NCT01713582
BI 894999	BRD4 (BD1), BRD3 (BD2)	Inhibits binding of BRD4 and BRD3 with acetylated histone proteins	Phase I	Advanced malignancies	NCT02516553

#### Succinylation-related inhibitors

Most methods targeting Ksuc and Kac overlap, as do their inhibitors or activators, except for the Ksuc-specific writer CPT1A. To date, only one specific inhibitor of CPT1A has been identified, ST1326, which inhibits cancer cell proliferation in AML. However, ST1326 acts through modulating FAO rather than inhibiting protein succinylation [371–373]. SIRT5 and SIRT7 are succinylation erasers. Therefore, pan-HDAC inhibitors also affect desuccinylation activity. Inhibitors specifically targeting SIRT5 and SIRT7 must be developed, given the critical role of succinylation regulation in cancer and the limitations of broad-spectrum HDAC inhibitors.

#### Myristoylation-related inhibitors

NMT1 and NMT2 are myristoylation writers. Compounds such as IMP-1088, IMP-366, and PCLX-001 are selective NMT inhibitors. These inhibitors are potential tumor suppressors, and the tumor-suppressive mechanisms of IMP-366 and PCLX-001 have been reported [374]. However, only PCLX-001 has advanced to Phase I clinical trials, in which it was administered for therapeutically managing B-cell non-Hodgkin's and advanced solid tumors. Subsequent animal studies and case reports corroborated the efficacy of PCLX-001 in inhibiting breast cancer and B-cell lymphoma [280, 292, 375]. However, evidence regarding the efficacy of PCLX-001 remains limited, and further studies are needed. SIRT6 and IpaJ function as erasers of lysine and N-terminal glycine myristoylation modifications, respectively. However, no targeted inhibitors have been developed to modulate the activity of SIRT6 and IpaJ, and thus regulate myristoylation.

#### Palmitoylation-related inhibitors

Although no specific ZDHHC inhibitors have been identified, 2-bromohexadecanoic acid (2-BP), cerulenin, and tunicamycin have been widely used as PAT inhibitors, with 2-BP reaching the clinical trial stage for several noncancer diseases. The mechanism of action of these PAT inhibitors is not understood, and clinical evaluations for their suitability as cancer treatments are lacking. Developing palmitoylated writer inhibitors is difficult due to the structural similarity of PATs. Yao et al. synthesized a peptide derived from PD-L1 and PD-1 that strongly inhibited PD-1/PD-L1 palmitoylation [376, 377]. However, this peptide competitively inhibits PD-1 palmitoylation and expression and cannot be strictly considered a PAT inhibitor. These findings provide insights for developing strategies targeting palmitoylation in cancer therapy.

Research investigating palmitoylated eraser inhibitors is still in the early stages. Palmostatin B and palmostatin M nonspecifically modify and inactivate the serine residues in APT1 and APT2 active sites [378, 379]. Inhibitor21 and inhibitor1 are specific and respective inhibitors of APT1 and APT2, respectively [380]. Inhibitors of PPT1, didemnin B, and the dimeric quinoline compound DQ661 hinder the growth of solid tumors and disrupt lysosomal function [381, 382]. ABD957, part of the ABHD17 family of depalmitoylase inhibitors, is highly selective and only partially affects NRAS palmitoylation but also impairs NRAS signaling and growth of NRAS-mutant AML cells [383]. However, none of these inhibitors has entered clinical trials for treating cancer.

**Fig. 5:**
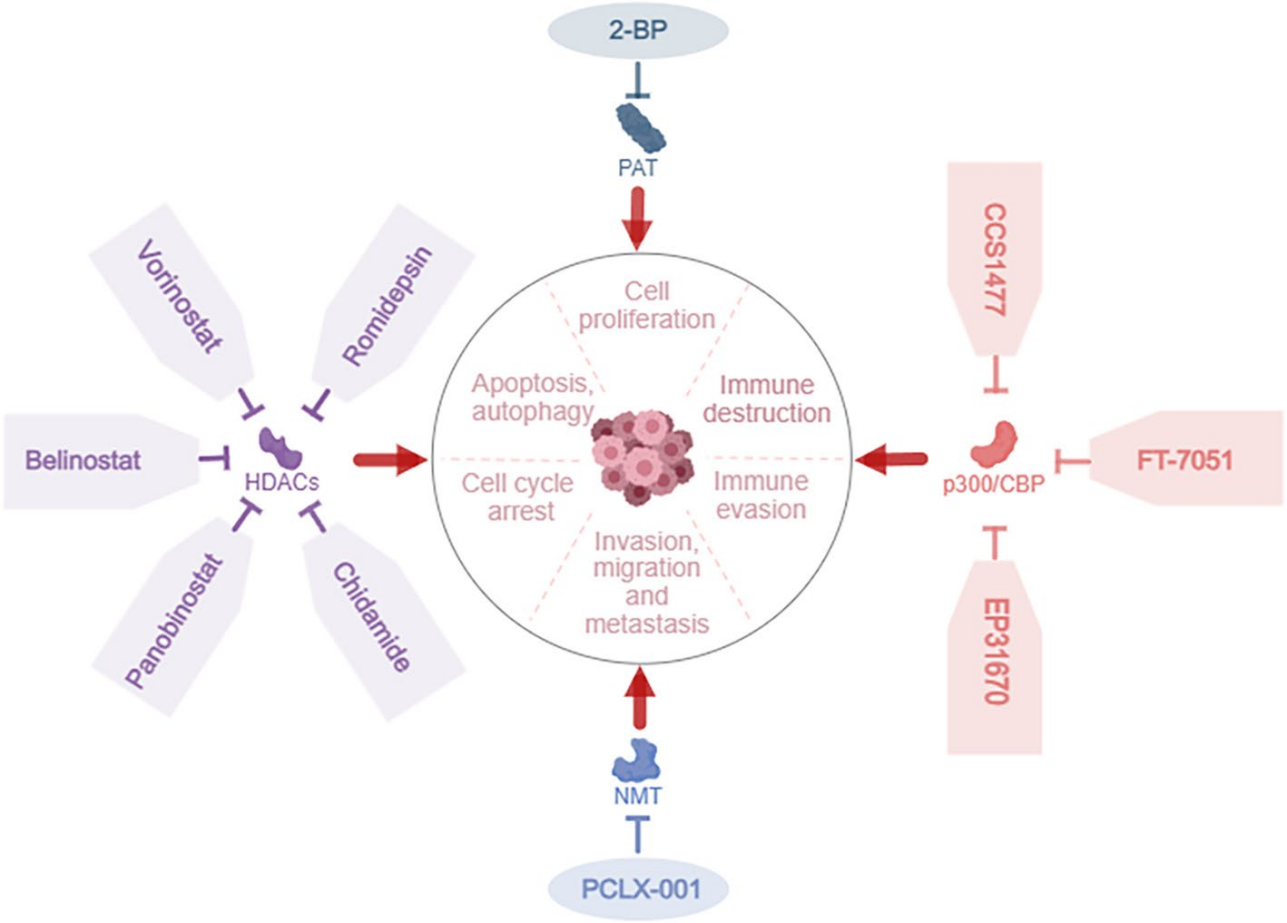
Therapeutic role of protein acylation inhibitors in tumors

The evidence collectively indicates that these proteolipid acylation inhibitors modulate their corresponding proteolipid acylation modifications during cancer progression by inhibiting their respective writers, erasers, and readers. This inhibition modulates various aspects of tumor progression, such as cell proliferation, autophagy, apoptosis, immune destruction, immune evasion, and tumor invasion (Fig. 5).

### Design of targeted drug delivery

Effectively treating cancer using anticancer drugs typically requires effective drug delivery systems [384]. Researchers are actively developing targeted nanodelivery systems in addition to the targeted drugs in clinical trials [385]. We summarize the strategies used for directly targeting protein acylation modification sites as well as the delivery systems used for pre-existing acylation modification inhibitors.

#### Nanomaterials directly targeting protein acylation modification sites

**Fig. 6:**
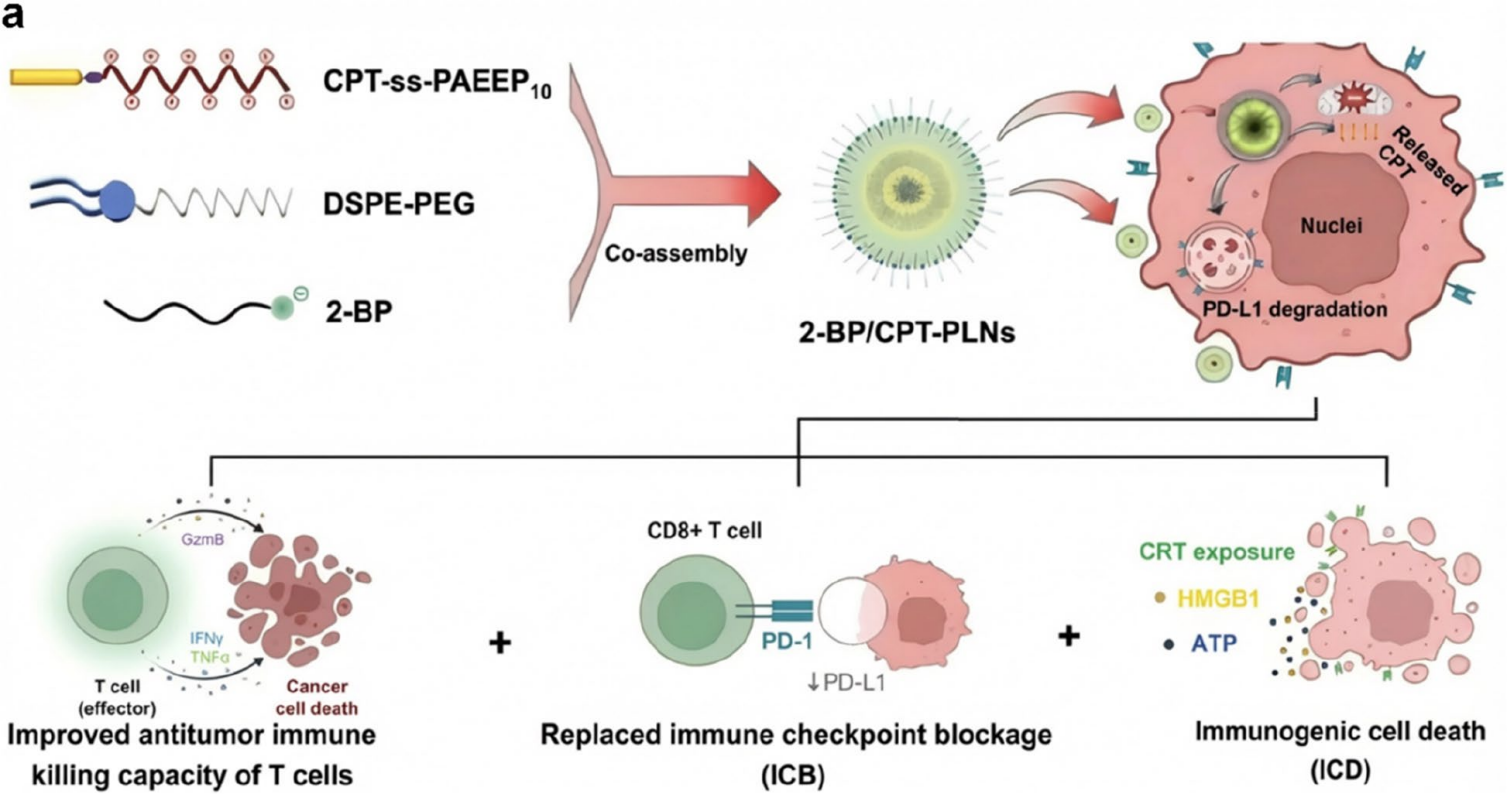
Nanomaterials that target protein acylation modification sites. Design and synergistic chemo-immunotherapeutic mechanisms of 2-BP/CPT-PLNs [388]

Few studies investigating nanomaterials have directly targeted protein–lipid acylation modification sites. No clinical trial has yet assessed 2-BP as a PAT in cancer therapy. However, the targeted delivery of 2-BP via nanomaterials has produced antitumor effects. The structural domain of PD-L1 in the cytoplasm is modified by palmitoylation, which prevents the ubiquitination and degradation of PD-L1, thereby hindering PD-L1 degradation. Tan et al. developed an irreversible palmitoylation inhibitor (2-BP/CPT-PLNs) assembled with CPT-ss-PAEEP10, DPSE-PEG, and 2-BP as hybrid polymer–lipid hybrid nanoparticles based on this finding [386]. These nanoparticles inhibit DHHC-mediated PD-L1 palmitoylation via 2-BP, promoting PD-L1 degradation and enhancing the efficacy of immune checkpoint blockade. These nanoparticles are expected to replace the alphaPD-L1 antibody with their efficient antitumor immunity through enhancing chemotherapy-induced immune cell death (Fig. 6a) [386, 387].

#### Delivery of protein acylation inhibitors

Many drugs targeting HDACs for the treatment of certain tumors have been approved or are currently in clinical trials. However, the disadvantages of these drugs, which include low solubility, short circulation time, and toxic effects, have hindered their use for treating solid tumors. Nanomaterials exhibit promise for addressing these limitations [389, 390]. Denis et al. synthesized a nontoxic pH-responsive drug delivery system to reduce the side effects of vorinostat and increase the activity of HDAC inhibitors in vivo [391]. Nanomaterials can improve the pharmacodynamics and pharmacokinetics of HDAC inhibitorss. Therefore, the composition or surface modification of nanomaterials could be changed to address these barriers. For example, hyaluronic acid (HA)-coated solid lipid nanoparticles (SLNs) are used to target the delivery of vorinostat to tumors through the specific binding of HA to the CD44 receptor. These nanoparticles exhibit high drug-loading capacity, sustained release patterns, high selectivity for potent oncological chemotherapy, and ability to maintain drug concentrations in the circulation over long periods, resulting in increased bioavailability [388]. The pharmacokinetic parameters of VRS in rats following intravenous administration depended on the formulation used. The Cmax values of the nanoparticle formulations (VRS-SLNs and HA-VRS-SLNs) were comparable to those of free VRS, but their pharmacokinetic profiles were substantially more suitable. VRS-SLNs exhibited 3.1-fold longer t1/2, a 1.8-fold higher AUC0-∞, and a 4.2-fold longer MRT (*p* < 0.05). These parameters were even higher for HA-VRS-SLNs versus VRS-SLNs, which had a 2.3-fold longer t1/2, a 2.0-fold larger AUC0-∞, and a 2.0-fold longer MRT (*p* < 0.05), indicating superior sustained release properties in rat models.

Targeted code delivery has also been developed for this field. Drugs targeting protein acylation modifications (e.g., HDAC inhibitors) are delivered along with anticancer drugs targeting other pathways in tumor cells (e.g., paclitaxel) to the tumor site via specially designed nanomaterials to increase the efficacy and synergy of combination chemotherapy. Wang et al. developed a smart biomimetic nanoplatform that combines targeted therapy with immunotherapy for treating metastatic triple-negative breast cancer [392]. This AFT/2-BP@PLGA@MD platform comprises nanoparticles encapsulated within tumor cell membranes. These nanoparticles demonstrated strong tumor-targeting ability, inhibited 4T1 tumor growth and metastasis, and effectively triggered an antitumor immune response in mouse models [392]. Li et al. developed a nanoplatform enabling temporal and spatial localization of anticancer drugs. This time-resolved nanoparticle produced strong antitumor effects in a mouse model of lung cancer compared to those produced by drugs not encased in nanoparticles or drugs modified with mesoporoussilica [393]. Exosome membranes were also detected using photoacoustic imaging for real-time diagnosis. The study established a new path for precisely delivering anticancer drugs such as suberoylanilide hydroxamic acid and suggested possibilities for future drug design and therapeutic protocols.

Nucleosomes or nucleosome arrays provide physiologically relevant substrates. These arrays compensate for the limitations of free peptides, which may not accurately represent chromatin biology, and offer a more effective platform for screening inhibitors or activators [41–43]. Scientists can more precisely control the timing and localization of drug release through in-depth analysis of drug delivery mechanisms, leading to the development and application of more personalized and effective therapies. This approach is expected to play a key role in future antitumor treatments by markedly improving therapeutic outcomes and enhancing the quality of life of patients [394, 395].

## Conclusions

In our review of acylation modifications, we considered protein modification in tumor metabolism. We classified the most commonly occurring types of acylation modifications based on those occurring on lysine and cysteine. We also described the various molecular mechanisms underlying these modifications to provide adjuvant therapeutic targets for clinical practice. Many challenges remain in developing targeted drug delivery strategies based on protein lipid acylation modification. For example, a key challenge involves designing drugs that selectively target abnormal levels of protein acylation without interfering with normal physiological acylation. Other features of the protein may need to be targeted using additional molecular modifications or specific properties of nanomaterials for precise targeting. Despite ongoing challenges, applying various functional modifications to nanomaterials, such as modifications targeting protein acylation, is a promising strategy for developing effective anticancer therapies.

## Supplementary Information

Supplementary Material 1.

## Data Availability

Not applicable.

## Abbreviations

PTMs: Post-translational modifications
PATs: Protein acyltransferases
DHHC: Asp-His-His-Cys
zDHHC: Zinc finger DHHC
KAT/KDAC: Lysine acetyltransferase/lysine deacetylase
NATs: N-terminal acetyltransferases
SIRT3: Sirtuin 3
MAPK: Mitogen-activated protein kinase
MKP1: MAPK phosphatase 1
MDM2: Mouse double minute 2 homolog
KDAC: Lysine deacetylase
TSA: Trichostatin A
IFI16: Interferon-gamma-induced protein 16
NCOR/SMRT: Nuclear receptor corepressors
COMT: Catechol-O-methyltransferase
NK: Natural killer
NKG2D: Natural killer group 2, member D
CTCL: Cutaneous T-cell lymphoma
PTCL: Peripheral T-cell lymphoma
HAT: Histone acetyltransferase
SDH: Succinate dehydrogenase
TCA: Tricarboxylic acid cycle
KGDHC: α-Ketoglutarate dehydrogenase complex
KAT2A/GCN5: Lysine acetyl-coenzyme A transferase 2A
H3K79: Histone 3 lysine 79
α-KG: Alpha-ketoglutarate
CPT1A: Carnitine palmitoyltransferase 1A
LDHA: Lactate dehydrogenase A
K47: Lysine residue 47
H3K122: Histone 3 lysine 122
PGAM1: Phosphoglycerate mutase 1
GLS: Glutaminase
SHMT2: Serine hydroxymethyltransferase 2
SDHA: Succinate dehydrogenase complex subunit A
PKM2: Pyruvate kinase M2
PRMT5: Protein arginine methyltransferase 5
G6P: Glucose-6-phosphate
CoAG6P: Coenzyme A glucose-6-phosphate
SUCLA2VDAC3: Succinate-CoA ligase ADP-forming beta Subunit-voltage-dependent anion channel 3
SREBP-1CoA: Sterol regulatory element-binding protein-1 coenzyme A
Fbw7 (FBXW7)SUCLA2: F-box and WD repeat-domain-containing 7 succinate-CoA Llgase ADP-forming beta subunit
PGC-1αSREBP-1: Peroxisome proliferator-activated receptor-gamma activator 1 alphasterol regulatory-element-binding protein-1
NF2Fbw7 (FBXW7): Neurofibromatosis type 2F-box and WD repeat-domain-containing 7
NMTsPGC-1α: N-myristoyltransferase peroxisome-proliferator-activated Receptor-gamma coactivator 1 alpha
PI3KNF2: Phosphoinositide 3-kinaseneurofibromatosis type 2
GAS41NMTs: Glioma-amplified sequence 41N-myristoyltransferases
TNF-αPI3K: Tumor necrosis factor-alpha phosphoinositide 3-kinase
NDPGAS41: N-myristolytic downregulated protein glioma-amplified sequence 41
NUPTNF-α: N-myristolytic upregulated protein tumor necrosis factor-alpha
mTORNDP: Mammalian target of rapamycin N myristolytic downregulated protein
AMLNUP: Acute myeloid leukemia N-myristolytic upregulated protein
ICAM-1mTOR: Intracellular adhesion molecule 1 mammalian target of rapamycin
FBXO4AML: F-box protein 4 acute myeloid leukemia
PPT-1/2ICAM-1: Palmitoyl protein thioesterase 1/2 intracellular adhesion molecule 1
ABHD10/17FBXO4: α/β Hydrolase-domain-containing protein 10/17 F-box protein 4
FASNPPT-1/2: Fatty acid synthase palmitoyl protein thioesterase-1/2
EGFRABHD10/17: Growth factor receptor α/β hydrolase-domain-containing protein 10/17
PD-L1FASN: Programmed death ligand 1fatty acid synthase
ACOX1EGFR: Acyl-coenzyme A oxidase 1epidermal growth factor receptor
GLUT1PD-L1: Glucose transporter 1 programmed death ligand 1
NRasACOX1: Neuroblastoma RAS viral oncogene homologacyl-coenzyme A oxidase 1
GARS1GLUT1: Glycyl-tRNA synthetase 1glucose transporter 1
EVsNRas: Extracellular vesicle neuroblastoma RAS viral oncogene homolog
CKAP4GARS1: Cytoskeleton-associated protein 4glycyl-tRNA synthetase 1
CD44EVs: Cluster of differentiation 44 extracellular vesicles
STINGCKAP4: Stimulator of interferon gene cytoskeleton-associated protein 4
VDAC2CD44: Voltage-dependent anion channel 2 cluster of differentiation 44
PCSK9STING: Proprotein convertase subtilisin/kexin type 9 stimulator of interferon genes
PTENVDAC2: Phosphatase and tensin homolog voltage-dependent anion channel 2
TeadsPCSK9: TEA domain transcription factor proprotein convertase subtilisin/kexin type 9
Tip60PTEN: TAT-interacting protein 60 phosphatase and tensin homolog
SRCTeads: Steroid receptor coactivator TEA domain transcription factors
ACAT1Tip60: Acetyl-CoA acetyltransferase 1TAT-interacting protein 60
CPT1ASRC: Carnitine palmitoyltransferase 1 asteroid receptor coactivator
PATsACAT1: Protein acyltransferases acetyl-CoA acetyltransferase 1
ShhCPT1A: Sonic hedgehog carnitine palmitoyltransferase 1A
HhatPATs: Hedgehog acyltransferase protein acyltransferases
APTShh: Acyl protein thioesterase sonic hedgehog
PPTHhat: Palmitoyl protein thioesterase hedgehog acyltransferase
ABHD17A/B/CAPT: α/β Hydrolase-domain-containing protein 17A/B/cacyl protein thioesterase
YEATSPPT: Yaf9, ENL, AF9, Taf14, and Sas5palmitoyl protein thioesterase
BETABHD17A/B/C: Bromodomain and extraterminal α/β hydrolase-domain-containing protein 17A/B/C
2-BPYEATS: 2-Bromohexadecanoic acidYaf9, ENL, AF9, Taf14, and Sas5
BET: Bromodomain and extraterminal
2-BP: 2-Bromohexadecanoic acid
