# Supplementary material for: Processes and therapeutic perspectives of acylation modifications of lysine and cysteine in tumors
Source: Cell Commun Signal. 2026 Feb 2;24:153. doi: 10.1186/s12964-026-02707-4 (PMC12952070; doi:10.1186/s12964-026-02707-4)
Supplement: Supplementary file 1 — Supplementary Material 1. [file 12964_2026_2707_MOESM1_ESM.pdf]

Table S1. Deacetylase inhibitors entering clinical trials.

Compound	Target	Mechanism	Clinical stage	Indications	ClinicalTrials.gov ID
Citarinostat	HDAC6	Suppress the function of HDAC6 and hinder cell growth.	Phase I	Multiplemyeloma	NCT02886065
AR-42	Pan HDACs	Reduce the levels of histone acetylation and diminish Akt pathway activity.	Phase I	Advanced cancer	NCT02886066
Tasquinimod	HDAC4	Function through the allosteric blockade of HDAC4 signaling pathways.	Phase II	Hepatocellular,Ovarian,Renal	NCT01743469
CXD101	HDAC class I	Suppress the enzymatic action of HDACs 1, 2, and 3.	Phase I	Cell and Gastric Cancers	NCT01977638
KA2507	HDAC6	Suppress the function of HDAC6 and exhibit anti-cancer properties as well as immune system regulation in preclinical studies.	Phase I	Advanced Cancer	NCT03008018

4-Phenylbutyric acid	Pan HDACs	Block the histone deacetylase activity and ER stress triggered by SiNPs within RAW264.7 cells.	Phase II, III	Lymphoma, adult solid tumors	NCT04531879
Tinostamustine	HDAC class I, II	Promote the addition of acetyl groups to histone or nonhistone proteins.	Phase I, II	Numerous Solid Tumors	NCT03345485
Domatinostat	HDAC class I	Stimulate the acetylation process of H3 histone proteins.	Phase II	GastrointEstinal Cancers	NCT03812796
CI-994	Pan HDACs	Suppress the enzymatic function of HDAC1, 2, 3, and 8, as well as curb cell proliferation.<br>.	Phase II	Advanced Myeloma	NCT00005624
Entinostat	HDAC1, 3	Suppress the function of HDAC1 and HDAC3, thereby triggering cell autophagy and apoptosis.	Phase II	Relapsed or Refractory Hodgkin's Lymphoma(ENGAGE-501)	NCT00866333
Givinostat	Pan		Phase	Duchenne	NCT03373969

	HDACs		III	muscular dystrophy (DMD)	
Abexinostat	Pan HDACs	It exhibits greater specificity for HDAC6 and HDAC8.	Phase III	Recurrent Glioma	NCT05698524
Tucidinostat	HDAC class I, HDAC10	Facilitate the acetylation process of the H3 protein.	Phase II	Metastatic Triple-negative Breast Cancer	NCT05390477
Panobinostat	Pan HDACs	Suppress the buildup of acetylated histone and nonhistone proteins, triggering autophagy.	Phase I, II	MDS/AML	NCT01451268
Belinostat	Pan HDACs	Suppress the buildup of acetylated histone and nonhistone proteins, triggering autophagy.	Phase I	Mild，Moderate and Severe Renal Impairment	Nct02679132
Romidepsin	Pan HDACs	Suppress the activity of HDAC1 and HDAC2, facilitating cellular apoptosis.	Phase II	Progressive or Relapsed Peripheral T-cell Lymphoma	NCT00426765
Vorinostat	Pan HDACs	Halt the accumulation of acetylated histone and nonhistone proteins.	Phase II	Lower Risk Myelodysplastic	NCT00486721
